# The lipoprotein biosynthesis pathway: key to OXA-mediated carbapenem resistance in *Acinetobacter baumannii*

**DOI:** 10.1128/aac.01099-25

**Published:** 2025-11-04

**Authors:** Jorgelina Morán-Barrio, Lucía Giacone, Luciano Brambilla, Carolina Fabbri, Alejandro M. Viale

**Affiliations:** 1 Instituto de Biología Molecular y Celular de Rosario (IBR, CONICET), Laboratorio de Resistencia a Antimicrobianos, Facultad de Ciencias Bioquímicas y Farmacéuticas, Universidad Nacional de Rosario 63031, Rosario, Argentina; Universita degli Studi di Roma "La Sapienza", Rome, Italy

**Keywords:** carbapenem-resistant *Acinetobacter baumannii*, lipo-β-lactamases, carbapenem-hydrolyzing class D β-lactamases, lipo-OXA carbapenemases, outer membrane vesicles, lipoprotein biosynthetic pathway

## Abstract

Carbapenem resistance in the gram-negative opportunistic pathogen *Acinetobacter baumannii* primarily stems from the overexpression of acquired class D serine β-lactamases, known as OXA carbapenemases. These enzymes exhibit weak carbapenemase activity and possess lipoprotein signal peptides. While the kinetic and structural aspects of OXA enzymes have been characterized, their biogenesis pathway has received little attention, despite potentially offering novel therapeutic targets. Here, we investigated the biosynthetic process of the OXA-58 carbapenemase in the model *A. baumannii* strain ATCC17978. [3H]palmitate labeling confirmed that the OXA-58 precursor is lipidated *in vivo*. Replacing the OXA-58 lipobox cysteine with alanine through site-directed mutagenesis demonstrated that, while the lipoprotein pathway is not essential for productive OXA-58 synthesis, it is crucial for achieving the high cellular OXA-58 levels *A. baumannii* needs to efficiently overcome carbapenem challenge. Lipidation significantly increased OXA-58 hydrophobicity, directing the carbapenemase to a membrane location, likely the outer membrane (OM), after periplasmic translocation. This specific localization is a critical step for accumulating the high periplasmic OXA-58 concentration necessary for carbapenem resistance. Furthermore, lipidation enabled the selective recruitment of OXA-58 into outer membrane vesicles (OMVs), revealing a novel disposal mechanism for surplus OXA-58 production. In conclusion, the *A. baumannii* lipoprotein biosynthetic pathway facilitates both the high periplasmic OXA-58 concentration essential for a more efficient carbapenem resistance and the accompanying selective removal of surplus OXA-58 production via OMV. These features were likely powerful drivers in the selection of the lipoprotein pathway for the overproduction of OXA carbapenemases among contemporary *A. baumannii* strains subjected to carbapenem challenge.

## INTRODUCTION

*Acinetobacter baumannii* (family *Moraxellaceae,* class *Gammaproteobacteria*) is a significant cause of healthcare-associated infections particularly affecting immunocompromised and severely injured patients (1, 2). The success of *A. baumannii* as a human pathogen is due in part to the remarkable ability of the species of the *Acinetobacter* genus for genetic exchange, environmental resilience, and genomic plasticity. This has led, under antimicrobial selection pressure, to the emergence of multidrug- resistant *A. baumannii* strains that include resistance to last-resort carbapenem β-lactams (CRAB). Developing novel therapeutic approaches against CRAB necessitates a detailed understanding of the mechanisms evolved by these strains to withstand carbapenem therapy and persist in vulnerable human hosts.

The primary cause of carbapenem resistance among CRAB strains is the overexpression of class D serine β-lactamases endowed with weak carbapenemase activity, known as carbapenem-hydrolyzing class D β-lactamases (CHDL), or OXA enzymes (1–5). These include the acquired OXA-58, OXA-23, and OXA-24/40, whose genes are carried by mobile genetic elements, and OXA-51, which is chromosomally encoded and serves as a hallmark of *A. baumannii* as a species. While these enzymes have been studied kinetically and structurally, less attention has been paid to their biogenesis pathway, which could also offer targets for drug design to control CRAB infections.

Like most β-lactamases produced in gram-negative bacteria, *A. baumannii* OXA enzymes are synthesized as preproteins with N-terminal signal peptides that direct them to the inner membrane Sec translocation machinery for subsequent export and fold within the periplasm. It is in the periplasmic compartment where they perform their protective functions, by hydrolyzing carbapenem and other β-lactam antibiotics (6–8). While most β-lactamases produced by gram-negative bacteria are located in the “soluble” periplasmic milieu, a few have been characterized as membrane-bound lipoproteins (9–14). Bacterial lipoproteins are a particular class of secreted proteins with no equivalents in eukaryotes, encoded by 1%–5% of bacterial genomes (15–18). Their precursors feature distinct N-terminal signal peptides that set them apart from conventional secretory proteins, including a four-amino acid motif (lipobox) carrying the invariant Cys residue destined for acylation during inner membrane translocation. Lipoprotein precursors are sequentially processed at this step by characteristic membrane enzymes. These include diacylglyceryl transferase (Lgt), lipoprotein signal peptidase II (Lsp), and apolipoprotein N-acyltransferase (Lnt). Lgt catalyzes the formation of a thioether linkage between the lipobox Cys and a diacylglycerol moiety, supplied by membrane phospholipids. This results in a modified signal peptide that Lsp then cleaves, exposing the N-terminal S-lipidated Cys to further N-acylation by Lnt, though the latter may be absent in some bacterial groups (17). Subsequently, unless a “Lol avoidance” signal (consisting of particular amino acids immediately after the lipobox Cys) is present, a Lol sorting pathway transfers the mature lipoprotein to the inner leaflet of the bacterial outer membrane (OM). There, it is anchored via the N-acylated Cys terminus, with the functional domain facing the periplasm.

Remarkably, the journey of certain β-lactamases may extend well beyond the periplasm. In various gram-negative bacterial species, including *A. baumannii*, β-lactamases belonging to different Ambler groups have been found to be recruited into released outer membrane vesicles (OMVs) (7, 13, 19–24). The OMV-mediated export of OXA enzymes has been proposed as a mechanism by which CRAB strains may indirectly enhance the pathogenicity of co-infecting susceptible bacteria by providing protection against carbapenem action (21).

OMVs are spheroid particles that bud and pinch off from the envelope of gram-negative bacterial species during growth (22, 23). OMVs are particularly enriched in OM constituents (hence their denomination), but they also contain components of other cell compartments including the periplasm. OMVs have been associated with many important functions related to cell viability and survival. These include waste disposal, envelope remodeling and maintenance, neutralization of antibiotics and other bactericides, nutrient scavenging, export of encapsulated bioactive molecules, cell-to-cell communication, and gene transfer (7, 21–23, 25–28). The detailed mechanisms underlying OMV biogenesis and cargo selection remain incompletely understood. These mechanisms likely vary depending on the specific bacterial species, the growth conditions, and the cargo considered (22, 23). In this context, for lipo-β-lactamases, this post-translational modification has been found necessary for their recruitment into OMVs (10–14, 29).

Various bioinformatic inferences indicate that all *Acinetobacter* OXA enzymes are equipped with lipoprotein transit peptides, sharply contrasting with their sequence-related OXA homologs in non-*Acinetobacter* taxa, which typically possess conventional signal peptides (13, 30, 31). The lipoprotein nature of *A. baumannii* OXAs could, therefore, explain their reported recruitment into OMVs (7, 21). Indeed, a recent site-directed mutagenesis study (13) provided evidence that lipidation of *A. baumannii* OXA-23 and OXA-24/40 actively promoted their membrane attachment and incorporation into OMVs.

We have previously characterized several plasmids from CRAB strains of our geographic region assigned to the ST15 (Pasteur scheme) (32–34). These plasmids carry a characteristic adaptive module bordered by XerC/D recognition sites containing the *bla*_OXA-58_ gene, which is prevalent in our area. In this module, the *bla*_OXA-58_ gene is embedded within a defective IS*Aba3*-based transposon accompanied with *araC* and *lysE* genes, with the latter being disrupted in our strains by an additional Tn*aphA6* transposon. Furthermore, *bla*_OXA-58_ is preceded by an IS*Aba825* element, whose insertion generated a strong hybrid promoter driving its overexpression (35).

Here, we investigated the effects of OXA-58 overexpression in *A. baumannii*, directed by recombinant and natural plasmids carrying the aforementioned IS*Aba825::bla*_OXA-58_ arrangement, using the ATCC17978 strain as a model. We experimentally demonstrate that OXA-58 is, indeed, synthesized as a lipoprotein in *A. baumannii*: Newly synthesized OXA-58 precursors could be labeled *in vivo* with [^3^H]palmitate, a post-translational modification that increased OXA-58 hydrophobic characteristics and directed it to a membrane destination, likely the OM. This attachment was necessary for *A. baumannii* not only to establish the high periplasmic concentration required to more efficiently overcome the carbapenem challenge but also for the selective disposal of the generated surplus lipo-OXA-58 into secreted OMVs.

These findings enhance our understanding of the mechanisms underlying the evolution of OXA-mediated carbapenem resistance in *A. baumannii* and reveal possible novel targets for the development of pharmaceuticals to control CRAB infections.

## RESULTS

### *In vivo* lipidation of the newly synthesized OXA-58 precursor in *A. baumannii*

**Fig 1:**
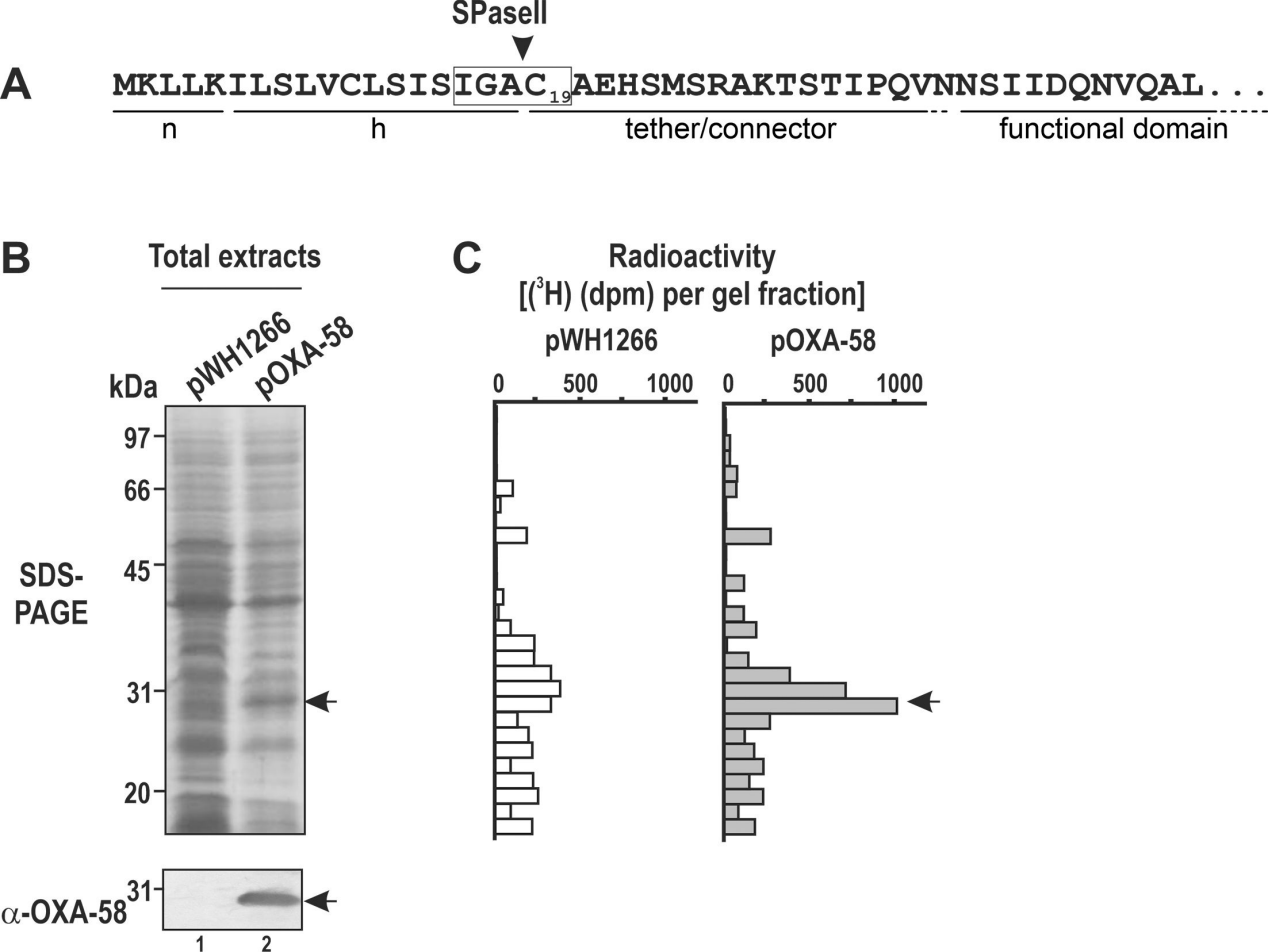
The *A. baumannii* OXA-58 is a lipoprotein. (**A**) N-terminal sequence regions of the OXA-58 precursor protein. The lipobox sequence containing the invariant Cys residue (C19) is boxed, and the predicted SPaseII cleavage site (SignalP-6.0 probability of 0.99) is indicated (▼). For other details, refer to Fig. S6. (**B**) SDS-PAGE/Coomassie blue protein staining and immunoblot analyses. Lane 1, total protein extract from ATCC17978 cells carrying the empty vector pWH1266. Lane 2, protein extract from cells overexpressing the OXA-58 precursor (pOXA-58). The cells were grown in BM2 minimal medium supplemented with 10 mM glutamate as the carbon source and pulse-labeled with [^3^H]palmitate before being collected and analyzed by SDS-PAGE. At the bottom, immunoblot analysis of the gel region containing the mature OXA-58 CHDL was performed with α-OXA-58 polyclonal antibodies. The migration and masses of the protein molecular markers are indicated at the left. (**C**) [^3^H]palmitoyl-protein in gel slices from (**B**). The closed arrows in B and C indicate the final OXA-58 position. See Materials and Methods for details.

Bioinformatic analyses inferred the presence of lipoprotein transit peptides in *Acinetobacter* CHDLs, unlike homolog OXA enzymes present in non-*Acinetobacter* bacterial taxa, which instead display conventional transit peptides (13, 30, 31). Specifically, the *A. baumannii* OXA-58 precursor shows the typical sectors displayed by lipoproteins (Fig. 1A). These include an N-terminal positively charged region (n), a hydrophobic helix region (h) ending in an IGAC lipobox (boxed) carrying the invariant Cys residue (C19) crucial for lipidation and subsequent SPaseII processing, and a tether or connector linking these regions to the conserved OXA functional domain. Of note, the OXA-58 transit peptide lacks the amino acid residues at positions + 2 to +4 (with respect to the lipobox Cys) that may provide Lol avoidance signals, leading to the retention of the lipoprotein in the inner membrane (15–18).

To test the prediction that the OXA-58 carbapenemase is lipidated *in vivo*, we first analyzed whether newly synthesized OXA-58 precursors could be radioactively labeled by applying a [^3^H]palmitate pulse to ATCC17978 cells transformed with plasmid pOXA-58 directing *bla*_OXA-58_ overexpression (Fig. 1). pOXA-58, which is a recombinant derivative of the *Acinetobacter* plasmid pWH1266 (36), carries a cloned 1.4 kbp amplified DNA fragment containing the complete *bla*_OXA-58_ gene precursor preceded by the 3´-terminal region of an IS*Aba825* element (35), hereby denominated ΔIS*Aba825::bla*_OXA-58_. Under the employed bacterial growth conditions, the externally added [^3^H]palmitate is incorporated into *A. baumannii* membrane phospholipids. Thus, providing the substrate for the subsequent Lgt-mediated introduction of [^3^H]palmitoyl-labeled diacylglyceryl moieties into the lipobox Cys of newly synthesized lipoprotein precursors during inner membrane translocation. We took advantage of the high constitutive synthesis of OXA-58 directed by pOXA-58 into ATCC17978 cells (Fig. 1B, see also below) to distinguish the [^3^H]-labeled OXA-58 from the labeled background of other lipoproteins synthesized *de novo*. Briefly, we labeled ATCC17978 cells carrying pOXA-58 (ATCC17978/pOXA-58) or pWH1266 (ATCC17978/pWH1266) with a [^3^H]palmitate pulse, subjected the labeled samples to SDS-PAGE, and compared the corresponding [^3^H]palmitoylated protein patterns (Fig. 1C). As shown in the figure, a considerable background of [^3^H]palmitoylated proteins was observed in both ATCC17978/pOXA-58- and ATCC17978/pWH1266-labeled cells, consistent with the findings of bioinformatic studies indicating the existence of around 68 different lipoproteins encoded in *Acinetobacter* genomes, covering a mass ranging from 10 to 110 kDa (15). As shown in Fig. 1C, a prominent [^3^H]-labeled protein band associated with the overproduced OXA-58 was differentially observed between ATCC17978/pOXA-58 and ATCC17978/pWH1266 cells, supporting the lipoprotein nature of OXA-58 synthesized in *A. baumannii*.

### Lipidation is not necessary for the productive synthesis of the OXA-58 CHDL in *A. baumannii*

**Fig 2:**
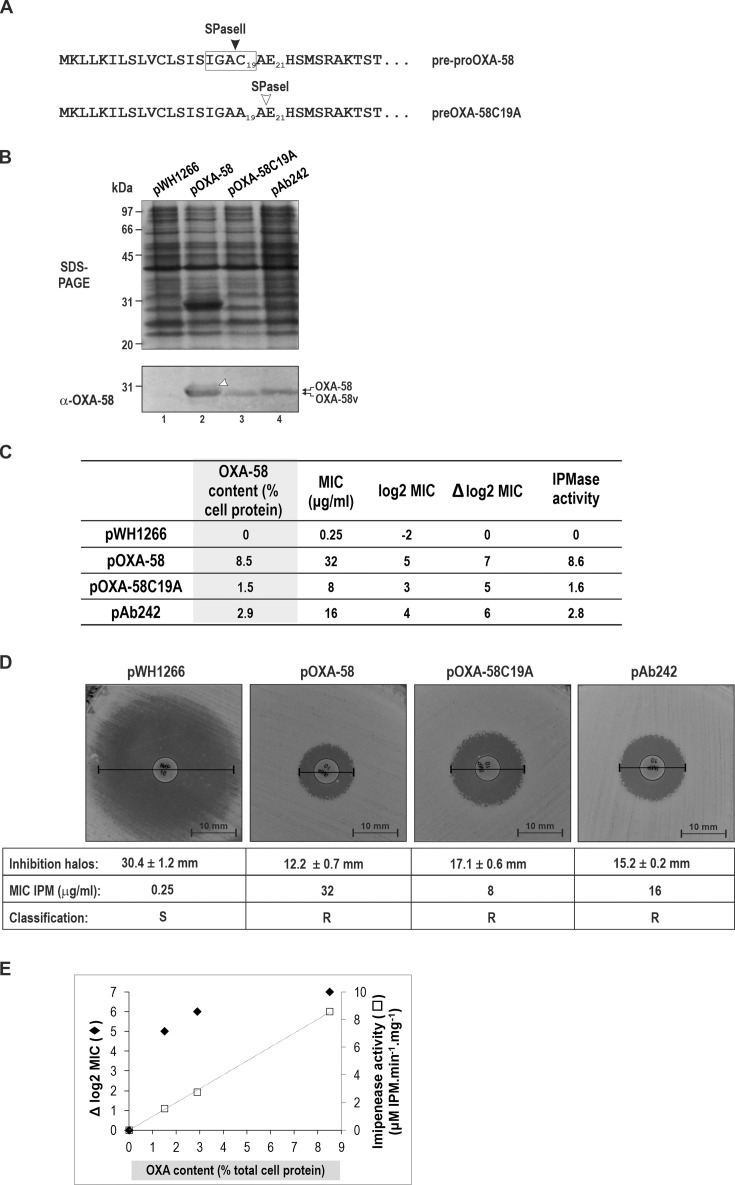
OXA-58 lipidation is not necessary for the acquisition of an active CHDL structure in *A. baumannii*. (**A**) N-terminal signal peptide sequences of the pre-proOXA-58 precursor (upper sequence) and the preOXA-58C19A mutant (lower sequence). Predicted signal peptidase II (SPaseII, close arrowhead) and signal peptidase I (SPaseI, SignalP-6.0 probability of 0.85, open arrowhead) are indicated. (**B**) SDS-PAGE and immunoblot analysis of OXA-58 expression. Total cell extracts from ATCC17978 cells carrying: lane 1, pWH1266 (empty vector); lane 2, pOXA-58; lane 3, pOXA-58C19A; lanes 4: pAb242. The final positions of OXA-58 and OXA-58v proteins in the immunoblot analysis are indicated (right margin). Pre-proOXA-58 is indicated with an open arrowhead in lane 2. (**C**) OXA-58 levels, IPM resistance, and IPMase activity in ATCC17978 cells harboring the indicated plasmids. Columns show: First column, OXA-58 contents as the percentage of total proteins based on densitometry; second column: MIC values for IMP (in μg/mL, determined by broth microdilution) for the corresponding cells; third column: log_2_MIC values; fourth column (Δlog_2_MIC), same as the third column, relative to cells carrying the empty vector; fifth column, IPMase activity (μM IPM hydrolyzed.min^-1^.(mg protein)^-1^) in the corresponding cell extracts. The OXA-58 protein content of ATCC17978/pOXA-58 cells was estimated by densitometric scanning in SDS-PAGE cells as 8.5%, while those of the other cells were calculated after densitometric scanning of the immunoblot membranes in B (bottom). (**D**) IPM inhibition halos on Mueller-Hinton agar obtained for ATCC17978 cells carrying the indicated plasmids. The MIC values for IMP in μg/mL are those informed in part C. IPM susceptibility (S) or resistance (R) is based on CLSI standards for clinical *Acinetobacter* isolates. (**E**) Correlation between OXA-58 levels and Δlog_2_MIC values (◆) or IPMase activity (□). See Materials and Methods for details.

Quantitative estimations based on densitometric scanning of Coomassie blue-stained gels indicated that the production of the acquired OXA-58 CHDL in ATCC17978/pOXA-58 cells reached approximately 8.5% of the total cell proteins, significantly surpassing the amounts of even the most abundant indigenous *A. baumannii* proteins (Fig. 2C). ATCC17978/pOXA-58 cells exhibited a clear carbapenem-resistant phenotype, as evidenced by increases in imipenem (IPM) MIC values (from 0.25 µg/ml in ATCC17978/pWH1266 cells to 32 µg/mL in ATCC17978/pOXA-58 cells) and reductions in IPM inhibition halos on Mueller-Hinton (MH) agar (from 30.4 mm to 12.2 mm, respectively) (Fig. 2D). Correspondingly, imipenemase (IPMase) activity values in crude ATCC17978/pOXA-58 cell extracts reached 8.6 μM IPM hydrolyzed.min^-1^.(mg total proteins)^-1^. In turn, negligible activity was detected in crude ATCC17978/pWH1266 cell extracts not producing OXA-58 (Fig. 2C).

Next, we analyzed whether a mutated pre-proOXA-58 version lacking the lipobox Cys19 could be productively synthesized in *A. baumannii*. Using site-directed mutagenesis, we replaced the Cys19 in the (WT) *bla*_OXA-58_ precursor encoded in pOXA-58 with an Ala, generating plasmid pOXA-58C19A (see Materials and Methods for details). SignalP-6.0 prediction analyses indicated that this replacement abolished SPII recognition but revealed the presence of an SPI recognition site located between amino acid positions 20 and 21 (Fig. 2A). These features allowed us to test whether a nonlipidated version of OXA-58, designated OXA-58v, could be synthesized, processed by the conventional Sec secretion pathway, translocated to the periplasm, and fold productively in this compartment. The predicted molecular mass of the mature OXA-58v is 29.4 kDa, i.e., slightly lower than the approximately 30.5 kDa mass predicted for a mature OXA-58 lipoprotein, which is two amino acids longer with a diacylglyceryl moiety covalently attached to its N-terminal Cys.

ATCC 17978/pOXA-58C19A cells effectively produced OXA-58v, as shown by SDS-PAGE/immunoblot analyses of the corresponding cell extracts (Fig. 2B, lanes 3). The OXA-58v protein produced in these cells exhibited slightly faster gel migration compared to the lipidated OXA-58, supporting differential processing of the corresponding precursors, as discussed above. Remarkably, OXA-58v levels in the cells reached only 1.5% of the total proteins, as evaluated by quantitative estimations based on densitometric analyses of the Coomassie blue-stained gels in combination with immunoblot analyses (Fig. 2B and C). These values represent approximately 18% of the levels found for the lipidated (WT) OXA-58 in ATCC17978 cells. Nonetheless, these levels permitted ATCC17978/pOXA-58C19A cells to acquire carbapenem resistance at the minimal CLSI cut-off value defined for *Acinetobacter* isolates (i.e., MIC for IPM of 8 µg/mL and inhibition halos on MH agar of 17.1 mm in diameter) (Fig. 2D). Expectedly, crude extracts from these cells displayed IPMase activity at lower levels (1.6 µM IPM hydrolyzed.min^-1^.mg^-1^) than those of ATCC17978/pOXA-58 cells (Fig. 2C).

The above results showed that, while the lipoprotein pathway was not strictly required for OXA-58 synthesis in *A. baumannii*, it significantly enhanced the cellular yields acquired by this CHDL. As indicated above, ATCC17978 cells expressing OXA-58v yielded approximately one-fifth of the OXA-58 levels observed in cells producing the (WT) CHDL (Fig. 2C). Given the near-identical sequences of plasmids pOXA-58 and pOXA-58C19A except for the introduced mutations, this difference in OXA-58 yields suggests that the lipoprotein pathway is considerably more efficient than the conventional Sec pathway when applied to OXA-58 production. Similar results were obtained when a different high-copy number plasmid vector, pVRL1 (37), was used as the cloning vector for the overexpression of *bla*_OXA-58_ or *bla*_OXA-58C19A_ (see Fig. S1 for details). pVRL1, despite sharing the same *Acinetobacter* replication module as pWH1266, carries an *aac1* (gentamicin) resistance cassette and a toxin-antitoxin addictive system (37). RT-qPCR analysis revealed comparable plasmid copy numbers in ATCC17978 cells (PCN) for pVRL1-OXA-58 (26.7 ± 2.2) and pVRL1-OXA-58C19A (29.0 ± 2.4), in concordance with the classification of pVRL1 as a high-copy number plasmid in *A. baumannii* (37). Therefore, the observed differences in OXA-58 yields between ATCC17978 cells carrying pVRL1-OXA-58 and pVRL1-OXA-58C19A cannot be attributable to a different PCN or to variations in the hybrid promoter sequences directing constitutive overexpression of the corresponding *bla*_OXA-58_ genes. This further supports the above conclusion that the lipoprotein pathway is more effective than the conventional secretion pathway for OXA-58 production in *A. baumannii*.

We also investigated OXA-58 levels and carbapenem resistance in ATCC17978 cells transformed with natural plasmids isolated from *A. baumannii* Ab242, a CRAB strain belonging to ST15 (Pasteur classification) isolated in our geographic area (32). One of these plasmids is a 24,808 bp bireplicon containing an adaptive module endowed with the IS*Aba825::bla*_OXA-58_ arrangement driving *bla*_OXA-58_ overexpression. The copy number of plasmids carrying this arrangement (pAb242) in these cells, determined by RT-qPCR, was 6.9 ± 0.9. This is approximately one-fourth of the values found for the pVRL1-based recombinant plasmids used above. It is worth noting that this PCN is within the range of the PCN values (between 5 and 6) determined for plasmids carrying the IS*Aba825::bla*_OXA-58_ present in the ST15 CRAB strains Ab242 or Ab825 of our collection (unpublished observations).

ATCC 17978/pAb242 cells exhibited (WT) OXA-58 levels of approximately 2.9% of total cell protein (Fig. 2C). This represented about one-third of the OXA-58 levels in ATCC17878/pOXA-58 cells and nearly twice those found in ATCC17878/pOXA-58C19A cells. This OXA-58 accumulation conferred ATCC17878/pAb242 cells a clear carbapenem-resistant phenotype, as judged by an IPM MIC of 16 µg/mL and inhibition halos on MH agar of 15.2 mm in diameter (Fig. 2D). Crude extracts of these cells also showed IPMase activity reaching 2.8 µM IPM hydrolyzed.min^-1^.mg^-1^ (Fig. 2C).

Analysis of OXA-58 contents, IPMase activity measured in crude cell extracts, and carbapenem resistance phenotypes (referred to as MIC for IPM) in the above-described ATCC17978 cells suggested that beyond a certain cellular OXA-58 threshold, further increases did not proportionally enhance IPM resistance (Fig. 2E). For instance, a 1.9-fold increase in OXA-58 contents from 1.5% of the total cell proteins (ATCC17978/pOXA-58C19A) to 2.9% (ATCC17978/pAb242) resulted in a similar 1.8-fold increase in IPMase activity (1.6 to 2.8 µM IPM hydrolyzed.min^-1^.mg^-1^) and a 1-fold log_2_ dilution increase in MIC (8 to 16 µg/mL) (Fig. 2C and D). However, a subsequent 2.9-fold increase in OXA-58 contents from 2.9% (ATCC17978/pOXA-58C19A) to 8.5% (ATCC17978/pOXA-58) of the total proteins resulted in a comparable 3.1-fold increase in IPMase activity (2.8 to 8.6 µM IPM hydrolyzed.min^-1^.mg^-1^), but only in a 1-fold log_2_ dilution increase in IPM MIC (16 to 32 µg/mL) (Fig. 2C and D). This implies that once a threshold level was exceeded, additional cellular OXA-58 contents did not significantly improve *A. baumannii* carbapenem resistance.

The apparent catalytic efficiency of enzymes inside living cells can be significantly lower compared to that under *in vitro* conditions, a situation attributed to substrate diffusion limitations caused by the macromolecular crowded intracellular environment (38). These limitations could be further amplified as the enzyme concentration rises due to the associated increases in the medium viscosity or if the substrate has to permeate the OM (39, 40). The latter is the case of the carbapenems in *A. baumannii*, which must traverse the strict OM permeable barrier of this organism via selective channels displaying saturation kinetics (41). Consequently, different *in vivo* diffusion barriers that restrict IPM access to the OXA-58 active site could explain the lack of correlation between the levels of this CHDL and IPM MICs in ATCC17978 cells beyond a certain high cellular OXA-58 threshold (Fig. 2E).

### Lipidation increases OXA-58 hydrophobicity

To assess how OXA-58 N-terminal Cys acylation influences its hydrophobic properties, we used a Triton X-114 (TX-114) phase separation assay designed to compare protein hydrophobicity (11, 42). This assay allowed us to compare the partitioning of WT OXA-58 with that of the mutated, nonlipidated variant OXA-58v, both generated in ATCC17978 cells as described above. TX-114 solutions have a relatively low clouding point at 22°C and separate into a detergent (Det) lipid phase and an aqueous (Aq) phase above this temperature. When applied to a biological sample, this separation results in an enrichment of hydrophobic proteins in the Det phase, while the hydrophilic proteins remain in the Aq phase.

TX-114 phase fractionation followed by SDS-PAGE and immunoblot analysis (Fig. S2) indicated a differential enrichment of the WT OXA-58 in the Det phase. In contrast, OXA-58v was predominantly recovered in the Aq phase (Fig. S2). These results demonstrate that *in vivo* OXA-58 N-terminal Cys acylation substantially increases the hydrophobicity of this CHDL, consistent with the situation expected for lipoproteins (16, 18).

### OXA-58 lipidation in *A. baumannii* promotes membrane association

**Fig 3:**
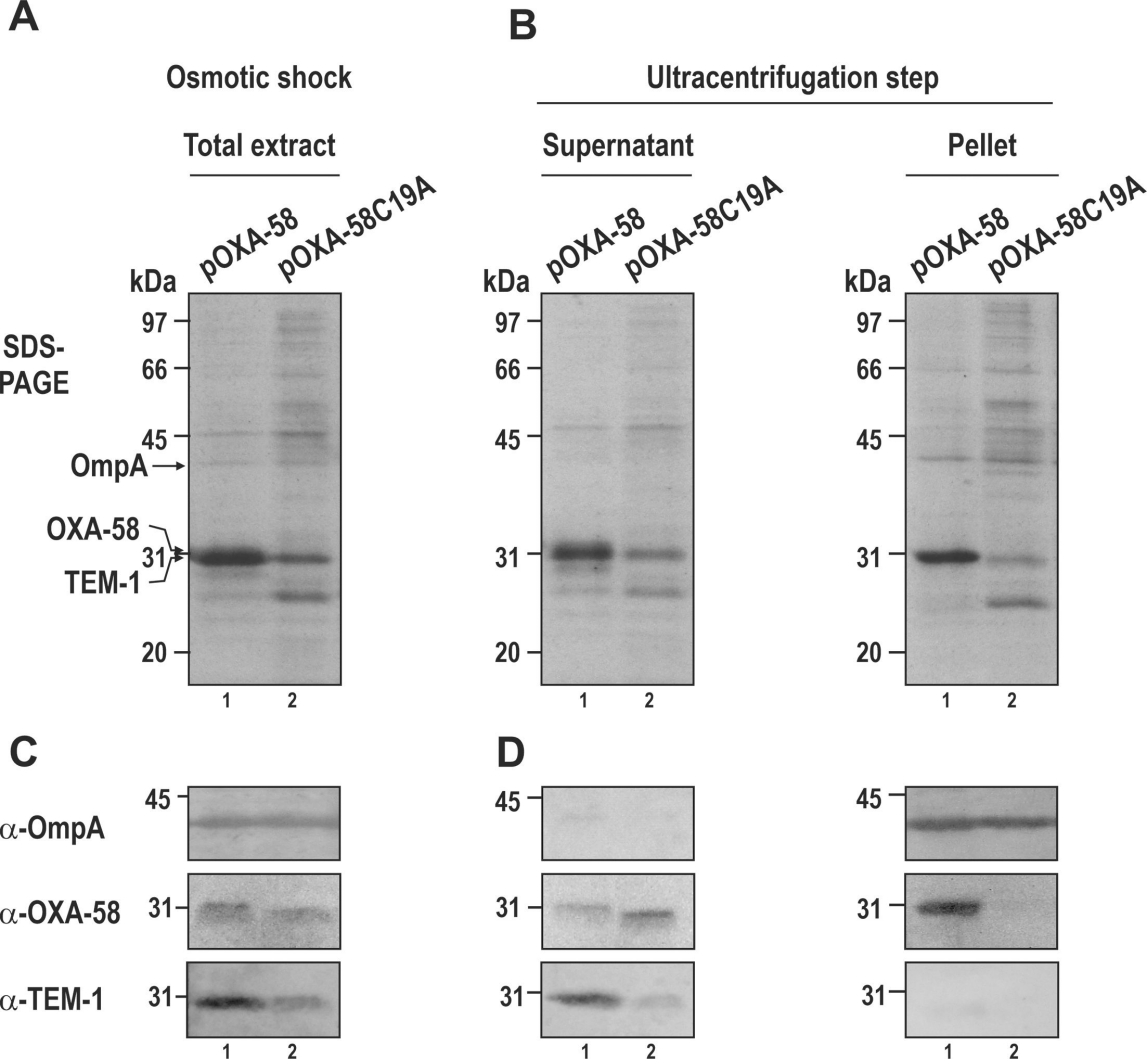
OXA-58 lipidation is required for membrane association. (**A**) SDS-PAGE analysis of periplasmic fractions obtained after osmotic shock. Periplasmic-enriched fractions from ATCC17978 cells expressing OXA-58 (pOXA-58, lane 1) or OXA-58C19A (pOXA-58C19A, lane 2). (**B**) Same, after subjecting the fractions from (**A**) to an ultracentrifugation step aimed to separate soluble proteins (supernatant) from membrane-associated proteins (pellet). (**C and D**) Immunoblot analyses of the gels shown in the upper figures separately revealed with antibodies against the intrinsic OM protein OmpA (α-OmpA), OXA-58 (α-OXA-58), or the periplasmic protein TEM-1 (α-TEM-1). Only the relevant portions are shown. The final positions of OmpA, OXA-58, and TEM-1 and those of the molecular mass markers are shown in the gels (left).

We next investigated whether the N-terminal Cys acylation of OXA-58 and consequent increase in hydrophobicity promoted its association with *A. baumannii* membranes following periplasmic translocation. To achieve this, we prepared periplasmic-enriched extracts from ATCC17978 cells carrying either pOXA-58 or pOXA-58C19A using a cold osmotic shock procedure (detailed in Materials and Methods). After osmotic shock, the crude periplasmic extracts obtained after removing the treated cells by centrifugation were analyzed by SDS-PAGE and immunoblotting using antibodies specific for OXA-58, the periplasmic TEM-1 β-lactamase, and the OM protein OmpA (Fig. 3).

This analysis indicated that these crude periplasmic extracts primarily contained TEM-1, yet also exhibited a noticeable presence of the OM material, evidenced by the co-occurrence of OmpA (Fig. 3A and C). The effective separation of the OM from the soluble periplasmic fraction was successfully achieved through ultracentrifugation at 300,000 × *g* for 3 h, as demonstrated by the predominant recovery of OmpA in the pellet fractions and TEM-1 in the supernatants following this step (Fig. 3B and D).

Immunodetection of OXA-58 in the supernatant and pellet fractions after ultracentrifugation revealed that the bulk of OXA-58 from ATCC17978/pOXA-58 cells co-sedimented with OmpA, thereby indicating membrane association for the lipidated form of OXA-58 (Fig. 3B and D, right panel, lane 1). Conversely, the nonlipidated OXA-58v was found in the soluble periplasmic fraction, alongside TEM-1 (Fig. 3B and D, left panel, lane 2).

Collectively, these results demonstrate that while OXA-58 lipidation is not strictly essential for inner membrane translocation or for its productive folding in the *A. baumannii* periplasm, it directs the CHDL to a membrane location subsequent to periplasmic translocation.

### Lipidation is required for OXA-58 recruitment into *A. baumannii* OMV

Similar to the case of OMV released by other bacterial species, lipoproteins are significant components of *A. baumannii* OMV, as shown by different proteomic analyses of vesicles released by both model strains and clinical isolates of this organism (19, 20, 24). Recent studies indicated that physical tethering of *E. coli* proteins to OM components favors their packaging into OMVs (28). Therefore, we investigated whether *A. baumannii* OXA-58 lipidation could explain its reported recruitment into *A. baumannii* OMVs (7).

**Fig 4:**
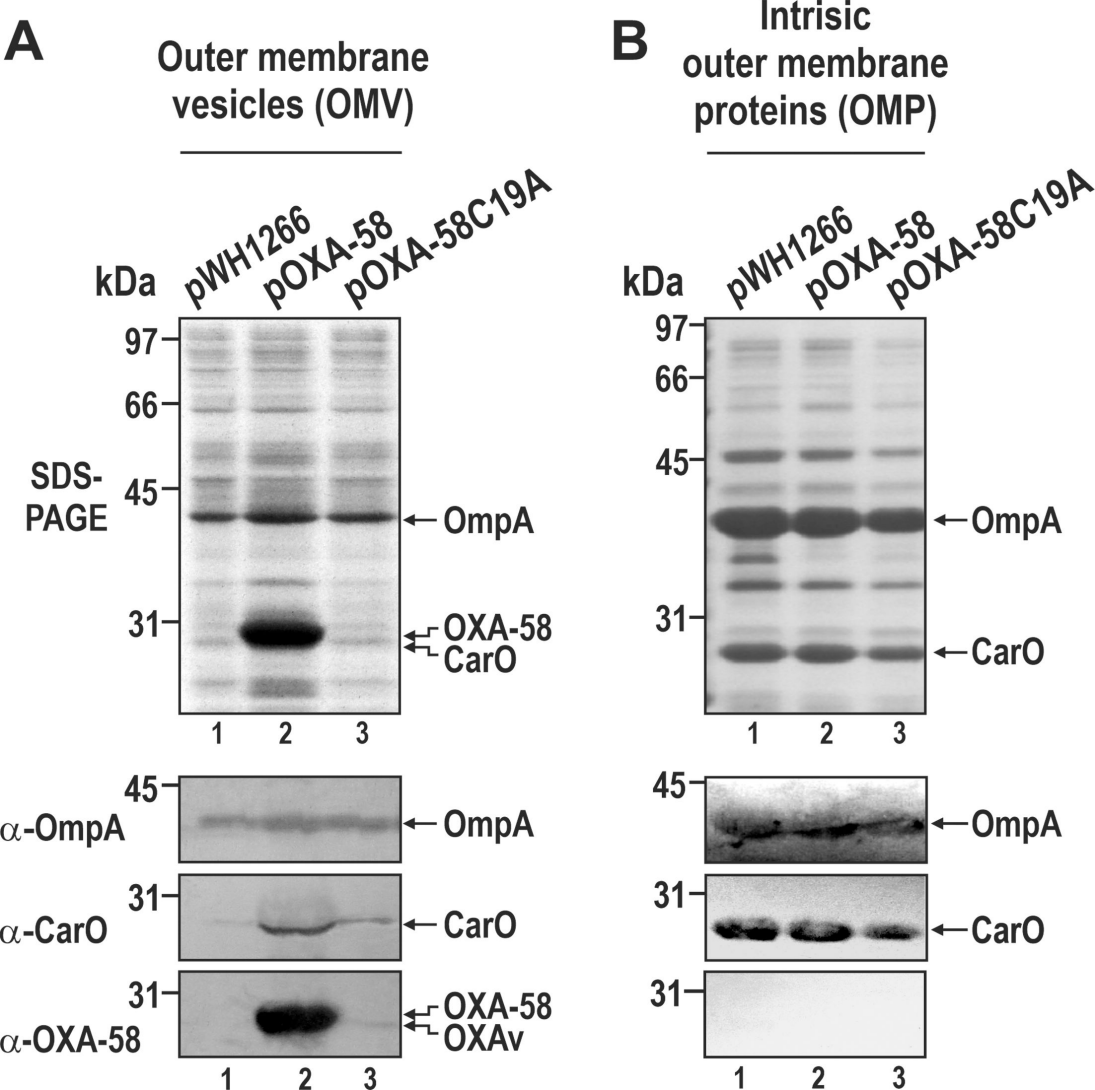
OXA-58 lipidation is required for its recruitment into *A. baumannii* OMVs. (**A**) SDS-PAGE and immunoblot analyses of OMVs shed by ATCC17978 cells harboring pWH1266 (lane 1), pOXA-58 (lane 2), or pOXA-58C19A (lane 3). Immunoblots were probed with α-OmpA, α-CarO, or α-OXA-58 antibodies. OMVs were purified from the cell-free culture media, resuspended in PBS, and analyzed by SDS-PAGE. (**B**) Same as in (**A**), but for the purified OM fractions from the same cells. The final positions of OXA-58, OXA-58v, OmpA, and CarO are shown at the right and molecular mass markers at the left.

OMVs were purified from ATCC17978 cells carrying pWH1266, pOXA-58, or pOXA-58C19A, grown in liquid culture without IPM supplementation. These OMVs were then analyzed by SDS-PAGE and immunoblotting to analyze the presence of OXA-58, as well as main intrinsic *A. baumannii* OMPs like OmpA and CarO (Fig. 4A). OM fractions from the same cells were also prepared using a N-lauroyl sarcosinate-based procedure (Fig. 4B), which removes peripheral proteins and lipoproteins, leaving only intrinsic OMP (43).

Analysis of the OM fractions indicated that OmpA and CarO represented the first and second, respectively, most abundant *intrinsic* OMPs of the *A. baumannii* OM (Fig. 4B). This is consistent with previous findings from our laboratory (41, 44). The protein profiles of OMVs (Fig. 4A) were similar across all tested cells but differed from the corresponding OMP profiles (Fig. 4B). OmpA in particular emerged as one of the most abundant proteins in the OMVs (Fig. 4A), agreeing with proteomic analyses of vesicles produced by other *A. baumannii* isolates (19, 20, 24). A notable exception was represented by OMVs shed by ATCC17978/pOXA-58 cells, which showed a massive and selective enrichment of lipidated OXA-58 (Fig. 4A, lane 2). In contrast, OMVs released by ATCC17978/pOXA-58C19A cells, producing the nonlipidated OXA-58v, contained barely detectable amounts of the soluble CHDL (Fig. 4A, lane 3).

The above results indicate that lipidation is essential for the recruitment of OXA-58 into *A. baumannii* OMV.

### Overexpression of OXA-58 increases the release of OMV selectively loaded with lipo-OXA-58 in *A. baumannii*

**Fig 5:**
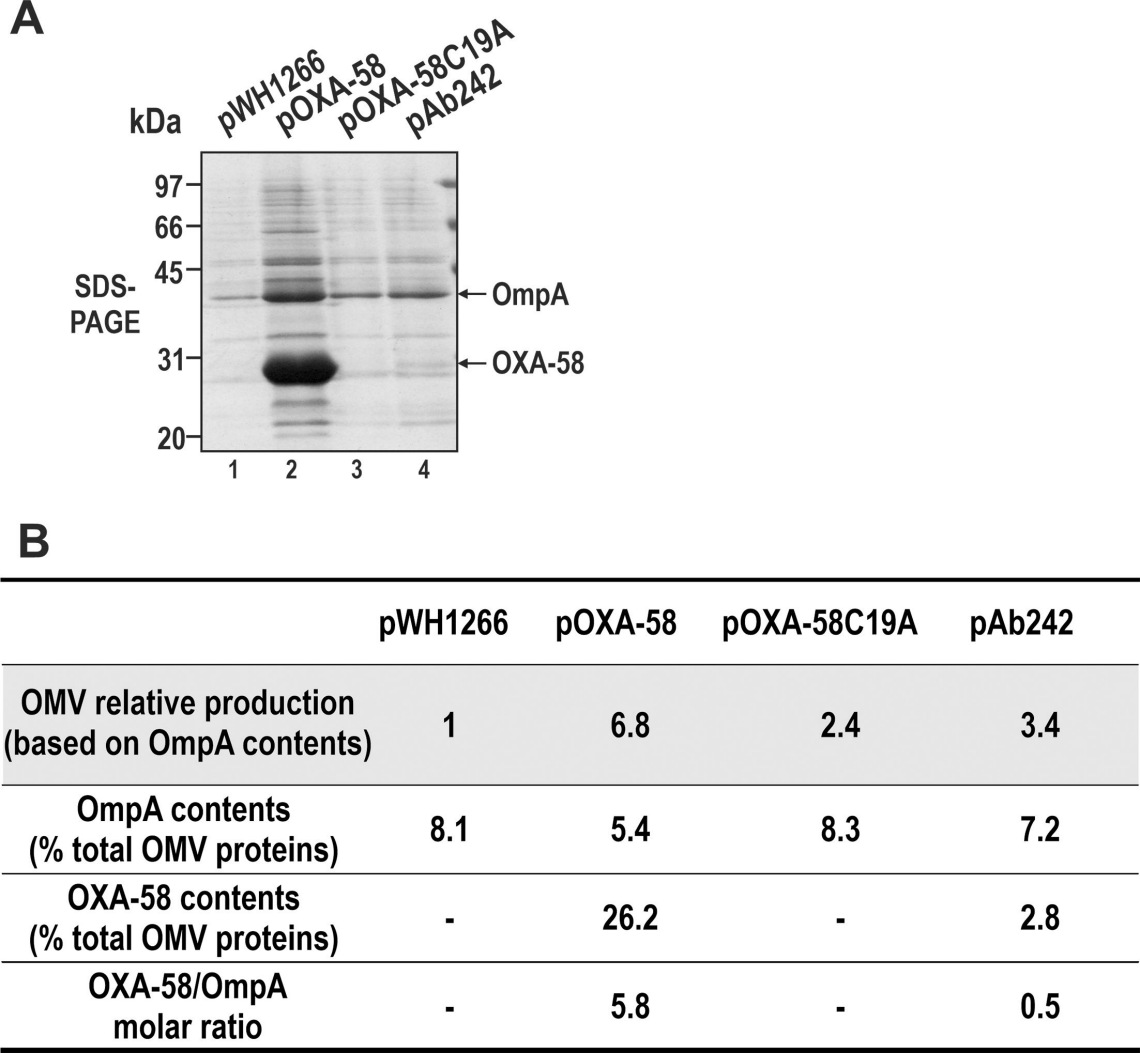
OXA-58 overexpression increases OMV release in *A. baumannii*. (**A**) SDS-PAGE analysis of OMVs isolated from cultures of ATCC17978 cells harboring pWH1266 (lane 1), pOXA-58 (lane 2), pOXA-58C19A (lane 3), or pAb242 (lane 4). Equivalent volumes of purified OMVs were loaded in the gels. OmpA and OXA-58 positions (as determined by a parallel immunoblotting, not shown) are indicated with arrows (right margin). Molecular mass markers are also indicated (left margin). (**B**) OMV production and OmpA and OXA-58 content comparisons. First row: relative OMV release by the indicated cells, measured as the OmpA content in each case (as judged by densitometry scanning) normalized to the OmpA value in OMVs released by ATCC17978/pWH1266 cells (Part A, lane 1). Second row: OmpA percentage in OMVs relative to total protein contents determined by densitometric scanning of the corresponding gel lanes in Part A. Third row: OXA-58 percentage in OMVs shed by the indicated cells relative to their total protein contents, in the corresponding lane. Fourth row: OXA-58/OmpA molar ratio. In OMVs from ATCC17978/pOXA-58 and ATCC17978/pAb242 cells. For calculations, the OXA-58 and OmpA molecular masses (mature proteins) were 30.5 and 36.2 kDa, respectively.

We next investigated the impact of OXA-58 overexpression on *A. baumannii* OMV production and the recruitment of this CHDL into these entities. OMVs were collected from OMV ATCC17978 cells carrying pWH1266, pOXA-58, pOXA-58C19A, or pAb242 grown at 37°C in LB liquid medium without IPM supplementation, resuspended in equal volumes of buffer solution, and analyzed by SDS-PAGE and immunoblot analysis with α-OXA-58 and α-OmpA antibodies (Fig. 5A). OmpA levels were quantified by densitometric analyses of the Coomassie blue-stained gels, and the relative differences in vesicle production between the different cells were estimated by normalizing the corresponding OmpA values to those of OMVs shed by ATCC17978/pWH1266 cells, which do not produce OXA-58 (Fig. 5B).

Based on OmpA comparisons, ATCC17978/pOXA-58 cells producing the lipidated OXA-58 to high cellular levels (8.5% of total cell proteins, Fig. 2) generated 6.8-fold more OMVs than ATCC17978/pWH1266 cells. These OMVs were highly enriched with lipo-OXA-58, estimated at 26% of their total protein content, with no appreciable changes in the relative contents of the other proteins that constitute these vesicles. The OXA-58 content on these OMVs substantially surpassed that of OmpA, with an estimated OXA-58/OmpA *molar* ratio of 5.8 (Fig. 5B).

ATCC17978/pAb242 cells, expressing lower cellular levels of OXA-58 (2.9% of total proteins, Fig. 2), also increased OMV production, estimated to be 3.4-fold higher than that of ATCC17978/pWH1266 cells that do not produce OXA-58 (Fig. 5B). However, the OXA-58 content in these OMV was only 2.8% of the total proteins, resulting in an OXA-58/OmpA molar ratio of 0.5 (Fig. 5B). Thus, an almost threefold increase in OXA-58 production (2.9% to 8.5% of total proteins, in ATCC17978/pAb242 and ATCC17978/pOXA-58, respectively, Fig. 2) in these cells led to a twofold increase in OMV release (based on OmpA relative comparisons, from 3.4 to 6.8), and an almost 12-fold increase in the loading of lipo-OXA-58 (on a molar basis, from 0.5 to 5.8) (Fig. 5B).

Consistent with Fig. 4, OMVs from ATCC17978/pOXA-58C19A cells contained negligible amounts of the soluble OXA-58v (Fig. 5A, lane 3). Notably, however, OMV release from these cells was still 2.4-fold higher than that from ATCC17978/pWH1266 cells (Fig. 5B).

We also investigated whether the overproduction of lipidated OXA-58 and its subsequent accumulation in the *A. baumannii* OM could negatively impact its permeability barrier functions. To address this, we assessed if OXA-58 overproduction in ATCC17978 cells enhanced the basal susceptibility of these cells to colistin by using a commercial colistin pre-diffusion assay (https://rosco-diagnostica.com/wp-content/uploads/98018-Print-Insert2016.pdf). We found no significant increases in colistin inhibition halos between ATCC17978 cells carrying either the pWH1266 plasmid vector or those directing OXA-58 overproduction (not shown). These observations suggest that the overproduction of the lipidated OXA-58, even at high levels, does not impact the functions of the *A. baumannii* OM permeability barrier. Although subtle effects could not be ruled out, the overall results above indicate the remarkable capacity of the *A. baumannii* OM to accommodate large quantities of lipo-OXA-58 molecules.

Overproduction of heterologous secretory proteins can trigger envelope stress responses in the bacterial host also, leading, among other symptoms, to increased OMV production (45–47). Our results indicate that OXA-58 overexpression increases OMV release in *A. baumannii*, regardless of whether the lipidated or the nonlipidated enzyme is overproduced. This indicates the triggering of stress responses in either of these cases. However, as shown above, it is notable that only the overexpression of the lipidated OXA-58 resulted in an increased release of OMV selectively loaded with this modified CHDL. The overall findings are, therefore, consistent with the idea that increased OMV release serves as a mechanism for *A. baumannii* to secrete and selectively discard the surplus lipo-OXA-58 production into the medium.

### OXA-58 localization in *A. baumannii* OMV and functional implications

The OXA-58 associated with OMVs released by ATCC17978/pOXA-58 cells was not accessible to degradation by proteinase K (Fig. S3A). SDS-PAGE/immunoblot analysis showed that OXA-58 persisted in these OMVs without signs of degradation for at least 120 min. Degradation occurred only after OMV disruption with SDS, indicating that the lipidated OXA-58 is located inside these entities, in agreement with previous reports (7). Consistent with both the final destination of most gram-negative bacterial lipoproteins and the proposed blebbing model for OMV biogenesis (16, 18, 22, 23), the recruited lipo-OXA-58 is likely attached to the OM inner leaflet of the *A. baumannii* cells.

Comparison of the IPMase activity of OMVs released by ATCC17978/pOXA-58 cells in the absence and presence of the non-ionic detergent Triton X-100 indicated a nearly twofold increase after vesicle dissolution and OXA-58 dilution into the assay medium (Fig. S3B). This increase in the activity after the discharge of the internally located lipo-OXA-58 into the medium supports the previously discussed existence (Fig. 2C) of *in vivo* barriers limiting either the full accessibility of the IPM substrate to the active site of OXA-58 or the efficient activation of the CHDL within the *A. baumannii* cell, particularly under the tightly packed conditions resulting from a high overexpression (Fig. 5).

It has been proposed that OXA-58 export inside OMVs could worsen the outcomes of polymicrobial infections by providing shelter to accompanying susceptible bacterial pathogens against carbapenem action (7, 21, 48). We observed that OMVs released from ATCC17978/pOXA-58 cells conferred protection against IPM to susceptible *A. baumannii* (Fig. S4A, lower disk) and *Escherichia coli* strains (not shown), evidenced by an increased growth of beneath susceptible bacteria near the disks loaded with OMV within IPM inhibition zones. OMV disruption with Triton X-100 significantly enhanced the protective effect only in the case of lipo-OXA-58-loaded vesicles (Fig. S5B, disk at the left). Conversely, OMVs from ATCC17978/pOXA-58C19A cells afforded no visible protection against IPM action (Fig. S4B), consistent with their less OXA-58v recruitment (Fig. 5). Remarkably, OMVs from ATCC17978/pAb242 cells, with much lower lipo-OXA-58 content as compared to those released from ATCC17978/pOXA-58 cells (Fig. 5), provided less protection, noticeable only after Triton X-100 addition (Fig. S4C).

It follows that OMVs loaded with lipo-OXA-58 released by CRAB strains can protect accompanying susceptible bacteria from imipenem action, but the afforded protection is strongly dependent on the OXA-58 overexpression levels in the *A. baumannii* cells generating the vesicles.

### Assessing the fitness impact of OXA-58 overproduction on *A. baumannii*

Maximizing the growth rate is a crucial fitness strategy for bacteria (40, 49). In this context, plasmid-driven *bla*_OXA-58_ overexpression in *A. baumannii* (Fig. 2) may impose a significant metabolic burden on the cells due to the increased demand of additional resources for OXA-58 production and concomitant OMV release associated with its disposal (Fig. 2 to 5). Metabolic burden typically manifests as reduced cell growth (47), which, in principle, would limit the competitive ability of OXA-58-overproducing CRAB strains to carbapenem-containing environments. Therefore, it is plausible that contemporary CRAB strains thriving in clinical environments─where carbapenem presence may be highly fluctuating or even absent for periods─have evolved trade-off strategies (49) aimed to maintain a basal cellular level of OXA CHDLs, sufficient to contest initial carbapenem challenge, at the lowest fitness cost.

The comparison of growth parameters between selected bacterial clonal lines in medium lacking antibiotic supplementation is employed as a simple and rapid procedure to estimate fitness effects of resistance plasmids (40, 50, 51). We decided to use this approach to assess the impact on *A. baumannii* fitness generated by plasmids directing *bla*_OXA-58_ overexpression used in this work. We used the pVRL1-based recombinant plasmids in this case (Fig. S2) as this vector contains a TA system ensuring persistence in the bacterial host (37), similar to the *A. baumannii* natural plasmid pAb242 (32) studied here.

The growth rates and lag periods of ATCC17978 cells carrying the pVRL1-based plasmids used here are presented in Table 1. In principle, the carriage of the “empty” pVRL1 alone resulted in a cost to ATCC17978 cells, evidenced by an approximately 16% reduction in the growth rate and a 1.2-fold increase in the lag period compared to nontransformed bacteria. ATCC17978/pVRL1-OXA-58 and ATCC17978/pVRL1-OXA-58C19A cells showed further reductions in the growth rate compared to ATCC17978/pVRL1 (24% and 13%, respectively; Table 1), indicating additional costs associated with the overproduction of either lipidated OXA-58 or its soluble OXA-58v version. However, while the lag period of ATCC17978/pVRL1-OXA-58 cells was not significantly different from that of ATCC17978/pVRL1, that of ATCC17978/pVRL1-OXA-58C19A was notably longer (1.8-fold; Table 1), even when the OXA-58v yields on these cells were lower (Fig. 2).

**TABLE 1:** Growth rates and lag periods of *A. baumannii* ATCC17978 cells carrying plasmids directing OXA-58 overexpression^*a*^

ATCC17978 transformed with:	Growth rate (min^-1^)	Lag period (min)
None	0.0089 ± 0.0005	48.9 ± 2.7
pVRL1	0.0075 ± 0.0002	57.0 ± 1.5
pVRL1-OXA-58	0.0057 ± 0.0001	56.6 ± 1.0
pVRL1-OXA-58C19A	0.0065 ± 0.0002	99.6 ± 3.0
pAb242	0.0072 ± 0.0001	49.0 ± 0.7

^*a*^ The indicated ATCC17978 cells were grown in LB liquid medium at 37°C without antibiotic supplementation. Growth was monitored by measuring Abs600 nm. For the growth rate and lag period calculations, see Materials and Methods.

Therefore, the observed reduction in growth rates for ATCC17978 cells (Table 1) indicates that the carriage of high copy-number plasmids directing overproduction of the lipidated OXA-58 or its soluble version generated fitness costs. Concerning lag, an extended lag period is considered to signal an adaptive bacterial response to stress (52). Thus, our results also strongly suggest that producing OXA-58 via the lipoprotein pathway imposes lower costs on the *A. baumannii* cell compared to the conventional secretion pathway followed by OXA-58v.

The analysis of the growth parameters of ATCC17978 cells carrying pAb242 also indicated a reduction in the growth rate (19%) compared to nontransformed cells (Table 1). This shows that the carriage of this natural plasmid and the consequent lipo-OXA-58 overexpression also generated a fitness cost to the cells. Notably, as also seen in Table 1, the lag period of ATCC17978/pAb242 cells was not significantly altered compared to nontransformed cells. This indicates that the overall costs of pAb242 carriage and associated OXA-58 overproduction were lower for *A. baumannii* than those generated by the high-copy number recombinant plasmids studied above.

Cells must balance the costs and benefits of protein expression to optimize fitness (40, 49). Our results show that an excessive OXA-58 production driven by high-copy number plasmids generated higher overall costs for *A. baumannii* than those resulting from pAb242, which has a much lower copy number, as shown above. Moreover, as shown in Fig. 2, excessive production of lipidated OXA-58 can even be superfluous for carbapenem resistance purposes. Therefore, the selection of moderate-to-low-copy-number replicons as carriers of IS::*bla*_OXA-58_ arrays most likely represents an *A. baumannii* trade-off strategy aimed at mitigating the overall costs of OXA-58 overexpression, while ensuring a sufficient level of CHDL to withstand potential carbapenem pressure, as suggested in Fig. 2E.

### Impact of *A. baumannii* OXA-58 overproduction in *E. coli*

Natural plasmids carrying modules with similar *bla*_OXA-58_-containing arrays are widespread among contemporary *A. baumannii* strains (see Fig. 4 in reference 32). Furthermore, *bla*_OXA-58_ has been more recently reported in clinical isolates of the *Enterobacteriaceae* (i.e., *Escherichia coli*, *Klebsiella pneumoniae*, and *Enterobacter* spp.) and the *Morganellaceae* (*Proteus mirabilis*) bacterial families ([53, 54], and references therein). These studies indicated the effective mobilization of *bla*_OXA-58_ genes to other human pathogens, some of which have been found to co-exist with *A. baumannii* in polymicrobial infections (48, 55–58).

We assessed whether an acquired *bla*_OXA-58_ gene could confer carbapenem resistance to an enterobacterial host. For this purpose, we examined the effects of plasmid-directed OXA-58 overproduction in the commonly employed *E. coli* DH5α strain. Initial transformation with the shuttle plasmid pVRL1-OXA-58 did not result in significant expression levels of OXA-58 in DH5α cells, as evaluated by immunoblotting analysis (not shown). Therefore, we generated pBAD-based plasmids cloning the *bla*_OXA-58_ coding region under an arabinose-induced promoter (see Materials and Methods for details). In DH5α cells grown in LB liquid medium with low arabinose concentrations (0.0001% wt/vol), immunoblot analysis revealed the production of detectable amounts of OXA-58 (Fig. S5A). Further attempts to increase the expression of *bla*_OXA-58_ using higher concentrations of arabinose (0.001%; 0.01% wt/vol) resulted in severe growth inhibition (Fig. S5B). The transformed *E. coli* cells grown at 0.0001% arabinose showed no significant changes in IPM MICs, although ampicillin MICs were found to be increased due to OXA-58 expression (Fig. S5C).

In summary, these results indicate that the lateral transfer of *bla*_OXA-58_ genes to *E. coli* can occur effectively and potentially provide increased resistance to a broad-spectrum penicillin such as ampicillin. However, the OXA-58 expression levels that *E. coli* could tolerate appeared insufficient to elicit a carbapenem-resistant phenotype, unlike the case of *A. baumannii*.

### Evolution of *Acinetobacter* OXAs

Bioinformatic analyses have revealed that all *Acinetobacter* OXA precursors possess lipoprotein transit peptides, a feature distinct from sequence-related OXA enzymes in non-*Acinetobacter* taxa, which exhibit conventional signal peptides (13, 30, 31). *Acinetobacter* OXAs also form a distinct cluster in phylogenetic trees, indicating closer sequence relatedness among themselves compared to non-*Acinetobacter* homologs (4, 31). This supports an early acquisition of an OXA gene by the *Acinetobacter* genus ancestor, followed by co-evolution with the host genomes during its diversification (4).

OXA-58, OXA-23, OXA-24/40, and OXA-51 are the primary CHDLs in *A. baumannii* (3, 5). Regarding their origins, while the chromosomally encoded OXA-51 is considered a hallmark of *A. baumannii* as a species, OXA-58, OXA-23, and OXA-24/40 are encoded on mobile genetic elements. Among them, OXA-23 likely originated in *A. radioresistens* (59), but the origins of OXA-58 and OXA-24/40 still remain unclear.

A detailed comparative analysis of the complete amino acid sequences of OXA-58, OXA-23, OXA-24/40, and OXA-51 precursors indicated identities that ranged between 50% and 66%, with OXA-58 representing the most divergent sequence among them (4). A more comprehensive sequence analysis (Fig. S6) indicated that their N-terminal regions encompassing the signal peptide and connector sequences showed the greatest variability in length, sequence, and lipobox location and composition (except for the invariant Cys). This indicates that the N-terminal sectors evolved at much faster rates when compared to the functional domains since these OXAs diverged from a common ancestor. As found in genome-wide analyses of secretory proteins with a common origin and function (60), this possibly reflects stronger selection associated with the adaptation to different hosts/niches during *Acinetobacter* diversification. It is, therefore, remarkable that a lipoprotein signal peptide has been strictly preserved among all of these OXAs, indicating that this trait provides a strong adaptive advantage in the context of *Acinetobacter* genomes.

## DISCUSSION

This study demonstrates that OXA-58 is synthesized as a lipoprotein in *A. baumannii* and that plasmid-directed overexpression and lipidation of this CHDL are crucial for achieving the high cellular levels required for clinical carbapenem resistance in this opportunistic pathogen. Bioinformatic analysis revealed that the N-terminal transit peptide of the OXA-58 precursor possesses the characteristic features that define bacterial lipoproteins, including an IGAC lipobox (Fig. 1). Consistent with this, [^3^H]palmitate labeling of *A. baumannii* ATCC17978 cells carrying plasmids directing overexpression of the OXA-58 precursor confirmed its *in vivo* post-translational acylation. Furthermore, a combination of site-directed mutagenesis, Triton X-114 fractionation, and cell fractionation assays showed that lipidation increased the hydrophobicity of OXA-58 and targeted this CHDL to a membrane location after periplasmic translocation.

Interestingly, OXA-58 lipidation is not essential for the productive production of this CHDL in *A. baumannii*. Replacing the Cys lipobox with Ala generated an OXA-58 variant incapable of lipidation but that could still translocate and undergo productive folding in the soluble periplasmic milieu. Indeed, this latter situation occurs in OXA homologs present in bacterial taxa other than *Acinetobacter*, which predominantly possess conventional signal peptides (13, 31). This restriction of lipoprotein signal peptides to *Acinetobacter* OXA enzymes raises questions about the selective pressure(s) driving the selection of this pathway for synthesis in this specific bacterial group. Given the low catalytic activity of OXA-58 toward carbapenems (3, 61), overexpression of *bla*_OXA-58_ is necessary for *A. baumannii* to acquire a carbapenem-resistant phenotype (35) (see also Fig. 2). As reported in this work, the lipoprotein pathway facilitates the acquisition of the high cellular levels of OXA-58 that allow *A. baumannii* to efficiently resist carbapenem challenge (Fig. 2 and Fig. S1). Furthermore, the lipoprotein pathway is not only significantly more efficient in terms of OXA-58 yields but also incurs lower overall fitness costs compared to the conventional Sec pathway, as evidenced by the significantly shorter adaptation period that lipoOXA-58-producing cells require to reinitiate growth (Table 1).

Several factors, some specific to the bacterial lipoprotein pathway and others to *A. baumannii*, could explain the increased accumulation of OXA-58 observed with *bla*_OXA-58_ overexpression (Fig. 2): first, the increased efficiency of bacterial lipoprotein synthesis, which has been attributed to the extremely rapid processing of lipoprotein precursors resulting from channeling interactions between membrane enzymes specific to this pathway (18). Second, the ability of lipoprotein precursors to utilize the signal recognition particle/YidC insertase pathway for Sec translocon targeting and insertion into the inner membrane (47, 62–67). This is expected to facilitate their rapid processing and translocation, especially under overproduction conditions, compared to precursors following the conventional secretion pathway. Third, specific features of *A. baumannii* such as a more flexible Lol trafficking system, which is apparently capable of transporting diacylated lipoproteins to the cell OM, which would facilitate the transit of lipidated OXA-58 to its final destination (17, 18). Fourth, the final attachment of lipo-OXA-58 to the inner leaflet of the *A. baumannii* OM, which positions this CHDL in a particularly protected compartment that favors the folding and stability of its functional domain (39, 68). This protected transit and membrane attachment also restricts nonproductive interactions of lipo-OXA-58 molecules with other macromolecules in the crowded periplasmic milieu, leading to off-pathways including aggregation (47) or interference with essential processes in the bulk periplasm (8, 69, 70). The above features, combined with the remarkable capacity demonstrated by the *A. baumannii* OM to accommodate a large number of lipo-OXA-58 molecules (Fig. 2 to 5), likely provide a reasonable explanation for the use and preservation of the lipoprotein pathway for OXA-58 overproduction in contemporary CRAB strains.

Beyond the reasons discussed above, OXA-58 lipidation also enables *A. baumannii* to dispose of the excess OXA-58 produced into the medium through OMVs (Fig. 4 and 5). The incorporation of undesirable components accumulated in the OM into secreted OMVs represents a mechanism used by many gram-negative bacteria to eliminate potentially harmful substances, as well as to maintain the structural integrity of this crucial permeability barrier (22, 23). In this context, physical tethering to OM components favors packaging into OMVs, with lipoproteins being preferentially recruited, especially upon stress (28). Indeed, our data indicate that lipidation of OXA-58 promotes its recruitment into *A. baumannii* OMV and that the release of vesicles selectively loaded with lipo-OXA-58 is enhanced by CHDL overexpression. This further supports the attachment of lipo-OXA-58 to the inner leaflet of the *A. baumannii* OM, consistent with both the final destination of most gram-negative bacterial lipoproteins and the blebbing model for OMV biogenesis (16, 18, 22, 23).

Consequently, the lipoprotein pathway for OXA-58 synthesis allows *A. baumannii* to both accumulate, under overexpression, this CHDL to levels required for effective carbapenem resistance and dispose of the excess OXA-58 produced by its selective secretion into OMVs. The combined features mentioned above most likely represented powerful drivers behind the selection and maintenance of the lipoprotein pathway for the overproduction of relatively inefficient carbapenemases such as OXA-58 in contemporary CRAB strains.

It is important to consider also that the entire process of overexpression, modification, translocation, and disposal of excess plasmid-encoded secretory protein, like OXA-58, could impose a significant metabolic burden on the *A. baumannii* cell, impairing overall fitness, especially in environments where carbapenem pressure is temporarily absent. Furthermore, our data indicated that such overproduction of OXA-58 may even be superfluous for carbapenem resistance purposes since increases beyond a high threshold of OXA-58 content did not translate into proportional gains in resistance (Fig. 2E). Therefore, the evolution of growth adaptability trade-off regulatory mechanisms would be expected among CRAB strains relying on *bla*_OXA_-overexpressing plasmids, aiming to balance maximal effective OXA carbapenemase production with minimal fitness costs (49). Based on our results, it seems likely that one such mechanism is to control the cellular dose of the IS*Aba825::bla*_OXA-58_ overexpression arrays, selecting replicons with low-to-moderate copy number (7–8, this work) as its carriers. Remarkably, similar PCN values (i.e., 5–6) were found for other plasmids with IS*Aba2::bla*_OXA-58_-containing arrays such as pA388 and pACICU1b, identified in CC1 and CC2 CRAB strains, respectively (71). The location of these arrangements within modules bordered by XerC/D sites, capable of mediating site-specific recombination events leading to plasmid shuffling events (32, 34), could facilitate the selection of novel replicons that allow an eventual *A. baumannii* host to better adapt to fluctuating carbapenem environments.

**Fig 6:**
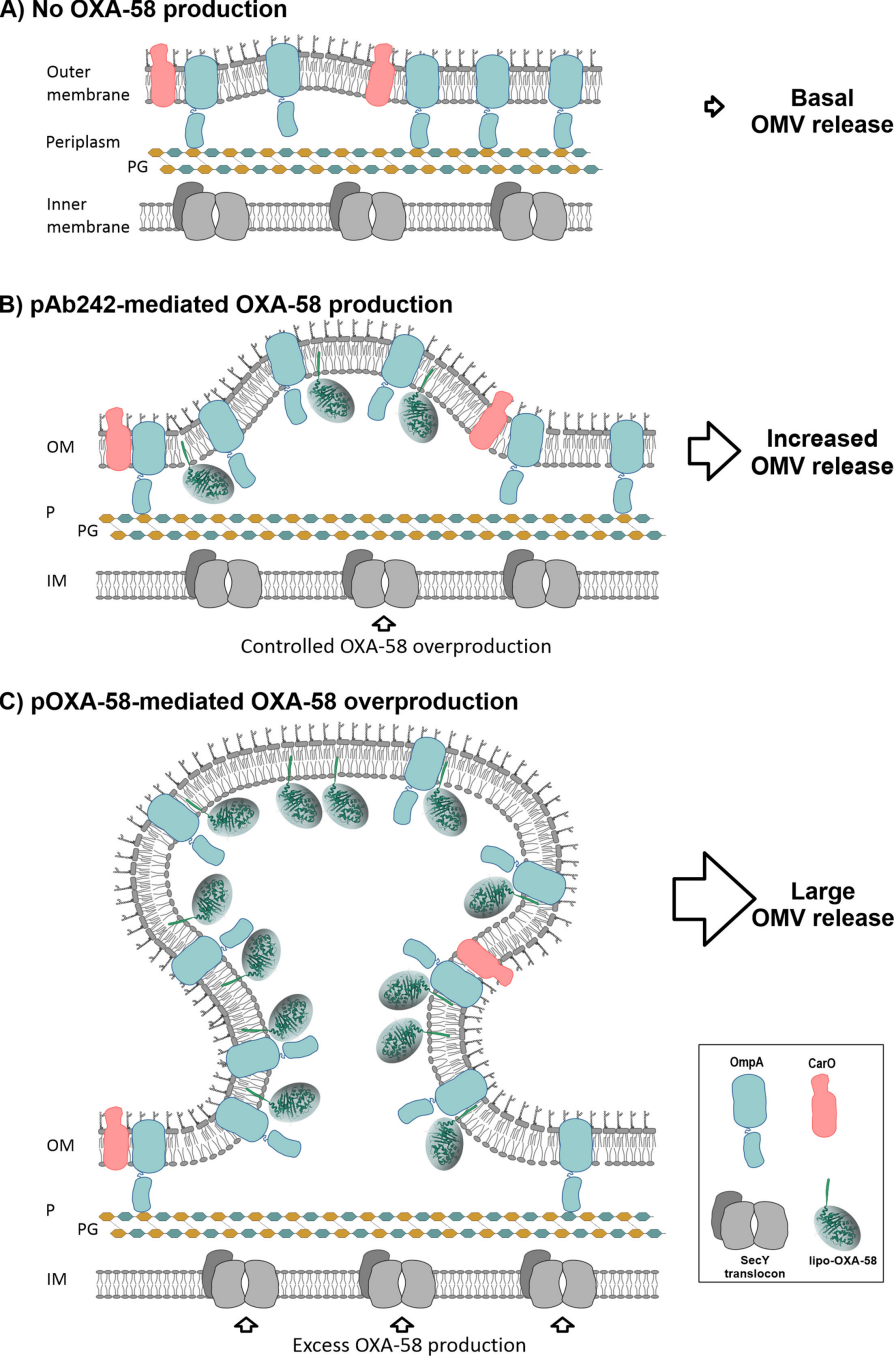
Proposed mechanism by which acquired OXA-58 overexpression in *A. baumannii* drives the selective recruitment of the CHDL into OMV. Panel (**A**) depicts the basal production of OMV. The *A. baumannii* envelope is stabilized by noncovalent linkages between the C-terminal periplasmic domain of OM-attached proteins such as OmpA, and the underlying peptidoglycan (PG). The number and specific location of OmpA-PG-stabilizing linkages determine which envelope areas are more or less prone to form OMVs, accounting for the basal release of vesicles. Panels (**B**) and (**C**) show how the acquired OXA-58 overproduction enhances OMV release. OXA-58 lipidation promotes its anchoring to the inner leaflet of the OM, interfering with the OmpA-PG stabilizing links and leading to increased OM detachment and the pinching off of OMV. The released OMVs are enriched in the membrane-attached CHDL, with the extent of this process depending on OXA-58 expression levels. For a more detailed explanation, see the text.

Our findings also suggest a mechanism by which OXA-58 overproduction can drive substantial increases in the production of OMV selectively loaded with this CHDL in *A. baumannii*. The consistent protein profiles of *A. baumannii* OMV carrying the different plasmids analyzed here, the presence of OmpA as their main constituent (Fig. 4 and 5), and the internal location of OXA-58 (Fig. S3) are all consistent with an OM blebbing model for vesicle biogenesis (22, 23, 28). In our proposed model (Fig. 6), lipidation of OXA-58 promotes its anchoring to the inner leaflet of the *A. baumannii* OM and its active recruitment into OMV. The gram-negative bacterial envelope is stabilized by linkages between a number of OM-attached proteins and the underlying peptidoglycan layer (PG) (22, 23, 72). In enterobacterial species, the primary stabilizing interactions depend on the Lpp lipoprotein, which is attached via its N-terminal lipophilic side to the OM and covalently bound at its C-terminus to the PG. However, in *A. baumannii*, which lacks Lpp homologs, this process relies on noncovalent linkages between the C-terminal periplasmic domain of OmpA and the PG (72, 73). It is generally assumed that OM microdomains form during gram-negative envelope biogenesis, in which the number and location of OM-PG-stabilizing linkages render different areas more or less prone to form OMVs (22, 23). This could explain the basal release of OMVs observed in *A. baumannii* cells not producing OXA-58 (Fig. 6A). In *A. baumannii* cells overexpressing the acquired OXA-58, the consequent accumulation of lipo-OXA-58 in these OM areas could interfere with the stabilizing OmpA-PG bonds. This interference would lead to increased OM detachment and the pinching off of OMV enriched in the attached CHDL depending on OXA-58 expression levels (Fig. 6, B and C). Notably, OXA β-lactamases seem particularly well-suited for this role. First, they exhibit similarly structured functional domains displaying common interacting surfaces with the C-terminal periplasmic region of *A. baumannii* OmpA (74–77). Second, they possess self-interacting surfaces capable of mediating the formation of dimers as well as other higher-order structures (3, 61, 76). Both features could lead, particularly under OXA-58 overexpression, to the formation of large protein clusters attached to the inner leaflet of the OM (39, 78) capable of driving increased OMV production. It is worth noting in the above context that the existence of particular domains in the functional region of OXA-58 that could trigger OMV formation was previously suggested by Liao et al. (7), based on experimental results obtained after overproducing a recombinant green fluorescent protein (EGFP) carrying the lipoprotein signal peptide of pre-OXA-58 in *A. baumannii*.

*A. baumannii* OMVs containing high levels of OXA CHDLs have been found to protect *in vitro* various susceptible bacterial species against carbapenem action (7, 13, 21) (see also above). Systematic reviews of literature indicate that approximately 20%–33% infections in which *Acinetobacter* (primarily *A. baumannii*) was identified also involved other bacterial pathogens (48, 55–58). However, the role of *A. baumannii* in these mixed infections remains unclear. Some studies reported no increased mortality compared to infections in which only this organism was identified (55, 57), while others observed an increased mortality only when other specific gram-negative pathogens were concomitantly present (48). *In vitro* studies indicate that *A. baumannii* can interact with other bacterial species in diverse ways, ranging from cooperative (13, 21, 79, 80) to highly antagonistic (81) depending on the strains involved. This variability may explain some of the above controversies, but it complicates predictions regarding the possible outcomes of mixed infections (48, 56). Our findings indicate that OXA-58 lipidation facilitates the disposal of the excess CHDL in overproducing CRAB strains via OMV. Whether the released vesicles can contribute to therapy failures by enhancing the pathogenesis of other species present in mixed infections is a threatening possibility that certainly warrants further investigation.

The overall findings of this work provide a better understanding of the mechanisms responsible for carbapenem resistance in *A. baumannii* and reveal potential targets for drug development aimed at controlling infections associated with this opportunistic pathogen.

## MATERIALS AND METHODS

### Plasmid vectors used for the expression of *bla*_OXA-58_ genes in *A. baumannii*

The *A. baumannii* ATCC17978 collection strain was used as the host for the various plasmids directing the expression of the WT *bla*_OXA-58_ gene or the mutated *bla*_OXA-58C19A_ precursor genes described in this work. pOXA-58 is a derivative of the *A. baumannii-E. coli* shuttle plasmid pWH1266, a recombinant plasmid vector based on the high-copy number replicon present in the *Acinetobacter* natural plasmid pWH1277 (36, 37). pOXA-58 carries a 1.4-kbp amplified DNA fragment, designated ΔIS*Aba825-bla*_OXA-58_, which contains the complete *bla*_OXA-58_ gene immediately preceded by the 3´-terminal region of an IS*Aba825* element that generated a strong hybrid promoter driving *bla*_OXA-58_ overexpression (35). The complete IS*Aba825-bla*_OXA-58_ arrangement forms part of a more complex adaptive module located in natural plasmids such as pAb242, carried by carbapenem-resistant *A. baumannii* clinical strains of our collection assigned to the ST15 (Pasteur scheme) (32, 34).

pOXA-58C19A is a pOXA-58 derivative in which the lipobox Cys19 residue of the OXA-58 precursor was replaced by an alanine (Fig. 2A). To introduce this mutation, the 1.4-kbp fragment containing the ΔIS*Aba825-bla*_OXA-58_ arrangement (see above) was first subcloned into the BamHI site of pBlueScript SKII (GeneScript), generating pBS-OXA-58 (~4.4 kbp). This plasmid was subsequently used as a template for a PCR-based site-directed mutagenesis, conducted essentially as described previously (82). We designed oligonucleotides OXA-58-Ala-Pst-Fw (5´-gcataagtattggggct**g*c****tgcag*agcatagtatgagtcgagc-3´) and OXA-58-Ala-Pst-Rv (5´-gctcgactcatactatgct*ctgca**g*****c**agccccaatacttatgc-3´), each complementary to the corresponding opposite strand region of the pBS-OXA-58 plasmid. The nucleotide changes (indicated in bold inside boxes) were designed to change the original Cys19 codon of the OXA-58 WT precursor gene to an Ala codon and also to incorporate a silent mutation (underlined *a* and corresponding *t* in the complementary strand) that generated a novel adjacent PstI restriction site, which was used for screening purposes. After PCR amplification, the resulting mixture was transformed into CaCl_2_-competent *E. coli* DH5α cells, and ampicillin-resistant colonies were selected on LB agar plates containing 50 µg/mL ampicillin. Plasmids were extracted from different colonies, screened for the presence of the newly introduced PstI site, and sequenced to identify one with the mutated *bla*_OXA-58C19A_ gene. The 1.4- kbp DNA fragment containing the desired mutation was then excised with BamHI and subsequently cloned into the equivalent site of pWH1266, generating pOXA-58C19A.

The *A. baumannii*/*E. coli* recombinant shuttle vector pVLR-1, carrying a gentamicin-resistant cassette as well as a toxin/antitoxin addictive module (37), was also employed for the generation of expression vectors directing production of the WT OXA-58 and the OXA-58C19A proteins in ATCC17978 cells. For this purpose, the 1.4-kbp BamHI fragments carrying the ΔIS*Aba825-bla*_OXA-58_ or its mutated ΔIS*Aba825-bla*_OXA-58C19A_ version (see above) were separately cloned into the equivalent site of pVLR-1, generating expression vectors pVLR-OXA-58 and pVLR-OXA-58C19A, respectively.

Natural plasmids extracted from the Ab242 *A. baumannii* strain (32) were also used to transform ATCC17978 cells in order to direct the expression of the (WT) pre-proOXA-58 precursor carbapenemase in this host.

### Plasmid vectors used for the expression of *bla*_OXA-58_ in *Escherichia coli*

The *E. coli* DH5α strain was used as a model to analyze OXA-58 production in an enterobacterial species. For this purpose, the *bla*_OXA-58_ gene was cloned into an *E. coli* pBAD expression vector under the control of an arabinose-induced promoter. The *bla*_OXA-58_-coding region was first PCR-amplified using as template plasmid pOXA-58 (see above) and oligonucleotides preOXANcoI-Fw (5´-tgtataccatggaattattaaaaatattgagtttag-3´) and preOXAEcoRI-Rv (5´-ctcggaattcttataaataatgaaaaacacccaacttatc-3´). This served to introduce NcoI and EcoRI restriction sites in the amplicon (boxed sequences), which was then digested with these enzymes and cloned into the equivalent sites of the pET28a (GeneScript), generating plasmid pET28a-OXA-58. This plasmid was digested with XbaI and EcoRI, and the fragment carrying the *bla*_OXA-58_ gene was purified and cloned into a pBAD expression vector (83) digested with NheI (which generates a site compatible with Xba) and EcoRI. These procedures yielded the *E. coli* expression vector pBAD-OXA-58 used in this study.

The *E. coli* DH5α strain (F^-^ *endA1 glnV44 thi-1 recA1 relA1 gyrA96 deoR nupG purB20 φ80dlacZΔM15 Δ(lacZYA-argF)U169, hsdR17(rm), λ^-^*) was used for both plasmid construction and expression purposes.

### Plasmid purification and transformation of ATCC17978 cells

The expression plasmids used in this study were extracted using an alkaline lysis procedure with the Wizard Plus SV Minipreps DNA Purification System (Promega, WI, USA). The quality of the extracted plasmids was routinely assessed by electrophoresis on 0.7% agarose gels, followed by ethidium bromide staining. Transformation of electrocompetent ATCC17978 cells was performed according to previously described procedures (35). For routine isolation of ATCC17978-resistant colonies after transformation, LB solid medium containing 1.5% (wt/vol) Difco Agar was used, supplemented with the following antibiotics when required: imipenem (IPM) 2 µg/mL; ampicillin (Amp) 300 µg/mL; kanamycin (Km) 50 µg/mL; gentamicin (Gm) 10 µg/mL.

### Bacterial culture conditions and IPM susceptibility tests

ATCC17978 cells harboring the various plasmids used in this study were grown under aerobic conditions at 37°C in liquid LB medium, with antibiotic supplementation as specified in the legends to Tables or Figures.

Carbapenem resistance in *A. baumannii* ATCC17978 and *E. coli* DH5α cells was assessed by determining the minimum inhibitory concentrations (MICs) for IPM, using the broth microdilution method, following the procedures recommended by the CLSI Performance Standards for Antimicrobial Susceptibility Testing M100, 30th edition, 2020. ATCC 17978 cells containing plasmids encoding the OXA-58 carbapenemase were thus considered to exhibit an acquired IPM-resistant (R) phenotype when their MIC was ≥ 8 µg/mL. Where shown, zones of inhibition determined by disk diffusion (disk containing 10 µg IPM) on Mueller-Hinton agar were also measured to provide a visual, comparative estimation of the various IPM-resistant phenotypes. In this case, a resistant (R) phenotype corresponds to a halo diameter ≤ 18 mm, following CLSI standards.

### ATCC17978 cells labeling with [^3^H]palmitate

ATCC17978 cells harboring pOXA-58 directing pre-proOXA-5 over-expression, or the pWH1266 cloning vector, were grown overnight at 30°C in BM2 minimal medium [62 mM potassium phosphate (pH 7.0), 7 mM (NH_4_)_2_SO_4_, 0.5 mM MgSO_4_, 10 µM FeSO_4_], supplemented with 10 mM potassium glutamate as the only carbon source (41) in the presence of 300 µg/mL ampicillin. The cultures were diluted 1:100 into 10 mL of fresh liquid medium, incubated at 30°C to an Abs_600_ of 0.3 under vigorous shaking, and labeled with 1 µCi per ml of [^3^H]palmitate (65 Ci/mmol, New England Nuclear) for 20 min under similar conditions. The reaction was terminated by adding trichloroacetic acid to a final concentration of 10% (wt/vol), and the tubes were transferred to an ice bath for 30 min. The precipitated material was recovered by centrifugation at 10,000 × *g* for 10 min at 4°C, and the pellets were rinsed twice with ice-cold acetone to remove the unincorporated [^3^H]palmitate. After air-drying, the pelleted material was resuspended in 0.1 mL of 20 mM Tris-HCl (pH 8.0), 50% wt/vol sucrose, 0.1 mM phenylmethylsulfonyl fluoride (PMSF), 0.1 mM EDTA, 1% wt/vol SDS. The mixture was cleared by centrifugation at 10,000 × *g* for 10 min at 4°C, and the total protein content of each sample was determined by a modified Lowry assay that incorporates 0.1% SDS (final concentration) to solubilize membrane proteins (44). Bovine serum albumin was used as a standard. Sample fractions containing 20 µg of the total protein were then subjected to SDS-PAGE*,* followed by Coomassie blue staining and destaining. For each sample, the gel lane was sliced in 30 equally sized horizontal portions, which were then placed into scintillation vials containing 1 mL of Eco-Lite scintillation counting solution (ICN Biomedical) to determine their [^3^H]-associated radioactivity using a Rackbeta 1219 Liquid Scintillation Counter (Pharmacia).

### Triton X-114 (TX-114) partition assay

The TX-114 partition assay (11, 42) was performed following described protocols with some modifications. Briefly, 0.5 mL of total extracts from ATCC17978 cells producing WT OXA-58 or OXA-58v were treated with 10% TX-114 (final concentration) in PBS buffer pH 7.2 at 4°C for 1 h. The samples were then incubated at 37°C for 3 min to induce microcondensation of detergent micelles, and the resulting phases were separated by centrifugation at 13,000 × *g* for 5 min at room temperature. The upper aqueous (Aq) phases and the lower detergent-enriched (Det) phases were separately collected for further processing. The Det phases were supplemented with ice-cold buffer to restore the initial volumes and incubated for 5 min on ice until a clear suspension was obtained. The tubes were transferred to 37°C for 3 min to induce a new phase separation and subjected to a further centrifugation at 13,000 × *g* for 5 min at room temperature. The Aq supernatant phases were removed, and the lower Det phases were supplemented with cold buffer to reach the original volumes, mixed, and incubated on ice until a clear suspension was formed again. The tubes were then centrifuged at 1,000 × *g* at 4°C for 5 min to remove any remaining particulate material, and the detergent-rich supernatants, enriched in hydrophobic proteins, were collected for further analysis. The Aq phases obtained after the first phase separation (see above) were supplemented with 10% vol/vol TX-114, and after a 5 min incubation at 4°C, the clarified suspensions were transferred to a 37°C water bath for 3 min to induce a new phase separation. After a further centrifugation step at 13,000 × *g* for 5 min at room temperature, the upper Aq phases were collected for further analysis. The protein material in the collected Aq or Det phases was precipitated by adding trichloroacetic acid (10% final concentration) and processed for subsequent SDS-PAGE and immunoblotting analyses.

### Preparation of periplasmic-enriched extracts of ATCC17978 cells

Various cell-fractionation protocols have been used to isolate *A. baumannii* periplasmic proteins, including chloroform-based, cold osmotic shock-based, and sucrose/lysozyme-based methods, all resulting in fractions enriched in periplasmic proteins with varying levels of contamination from the envelope and the cytoplasm ((84) and references therein). After evaluating different methods, we selected a cold osmotic shock procedure, complemented with an ultracentrifugation step of the extracts, as it better suited our objectives. Briefly, ACTT17978 cells harboring the plasmids indicated in figures or legends were cultured at 37°C in 30 mL of LB liquid medium until the stationary phase, collected by centrifugation at 1,500 × *g* for 20 min at 4°C, and rinsed with 20 mM Tris-HCl (pH 8.0), 150 mM NaCl. The collected cells were resuspended in 1 mL per g of fresh weight of ice-cold 20 mM Tris-HCl (pH 8.0), 50% (wt/vol) sucrose, 0.1 mM EDTA, and 0.1 mM phenylmethylsulfonyl fluoride (PMSF), and incubated under gentle agitation in an ice bath for 1 h. Ice-cold distilled water was then added in a proportion of 10 mL per mL of cell suspension, followed by a further incubation for 1 h under similar conditions. After removing the osmotic shock-treated cells by centrifugation at 8,000 × *g* for 10 min at 4°C, the clarified supernatants were subjected to ultracentrifugation at 300,000 × *g* for 3 h at 4°C to remove membrane-associated material. The supernatant and pellet fractions obtained after ultracentrifugation were separated and analyzed for total protein contents, SDS-PAGE, and immunoblotting. The rabbit antisera used for immunoblotting included the following: α-TEM-1 β-lactamase for a periplasmic protein encoded in plasmid pWH1266 (used here as vector for cloning purposes, Hunger et al. 2009); α-OmpA against the major intrinsic OMP of the *A. baumannii* OM (41, 44); and α-OXA-58, as analyzed in this study.

The total protein content of the different fractions was estimated by a modified Lowry assay that incorporates 0.1% SDS (final concentration) to solubilize membrane proteins (44). The samples were stored at −20°C until SDS-PAGE and immunoblot analyses, as described below.

### Isolation of OM fractions

The isolation of OM fractions of the different bacterial strains studied here was conducted using the N-lauroyl sarcosinate procedure, as previously described (41, 44). The total protein content of the samples was measured by a modified Lowry assay, as described above.

### Preparation of OMVs

OMVs were collected from culture supernatants of ATCC17978 cells transformed with the plasmids indicated in the legends to Tables or Figures, following reported procedures (7, 21). Briefly, 30 mL of LB broth was inoculated with 0.1 mL of an overnight culture of the cells under study and incubated at 37°C for 16 h with gentle shaking. The cells were then pelleted by centrifugation (8,000 × *g* for 12 min at 4°C), and the supernatants were filtered through a 0.22 µm pore-size filter membrane (Millipore Corporation, Bedford, MA). The filtered suspension was then subjected to ultracentrifugation at 300,000 × *g* for 2 hours at 4°C, and the pellet containing OMV was resuspended in 0.2 mL of PBS, pH 7.4. The protein content of the obtained OMV was determined using the Lowry modified assay mentioned above, and the samples were stored at −80°C for further analyses.

For quantification purposes and comparison of OMV yields in the analyzed (Fig. 5) *A. baumannii* cells, established procedures were used (85). Briefly, OMVs were prepared from a culture volume equivalent to 3 × 10^10^ colony-forming units (CFU) of the analyzed cells and resuspended in 0.2 mL of PBS as described above. Aliquots of equal volume of each OMV preparation were analyzed by SDS-PAGE/Coomassie blue staining, followed by OmpA quantification by densitometry in the different samples. Subsequently, relative OmpA values were calculated compared to a designated lane standard and used to infer OMV yields among the different analyzed cells.

The quality of the purified OMV was evaluated by subjecting an aliquot to proteinase K-agarose (Merck) digestion, as described below. As seen in the example provided in Fig. S3, no substantial modification to the OMV protein pattern was detected after 120 min of proteinase K treatment. This indicated no substantial levels of external contamination with attached (non-OMVs) proteins resulting from bacterial lysis during cell growth or manipulation (85). This OMV purification grade was considered sufficient for the purposes of this study.

### Determination of imipenemase activity on crude cell extracts of ATCC17978 cells

The IPMase activity was measured at 30°C by monitoring IPM hydrolysis at 300 nm using a Carry 60 UV-Vis Spectrophotometer (Agilent Technologies). The reaction mixture consisted of 50 mM phosphate buffer (pH 8.0), 100 µM IPM, 10 mM NaHCO_3_, and 20 µg total protein from cell extracts (41).

### SDS-PAGE and immunoblot analyses

The protein profiles of the different fractions were analyzed by SDS-PAGE using 12% (wt/vol) polyacrylamide gels, essentially as previously described (41). For immunoblotting, proteins were transferred from the gels to PVDF membranes in blotting buffer supplemented with 0.1% SDS.

The use of rabbit polyclonal antibodies against *A. baumannii* OmpA and CarO was described in previous works (41, 44). For the production of rabbit polyclonal α-OXA-58 antibodies, the OXA-58 antigen was electroeluted from gel slices after preparative SDS-PAGE loaded with OMV purified from ATCC17978/pOXA-58 cells. In turn, TEM-1 was purified to homogeneity from periplasmic extracts of *E. coli* DH5α/pWH1266, following previously described procedures (6) and used to generate anti-TEM-1 rabbit antibodies.

Quantitative evaluation of specific band intensities in Coomassie blue-stained gels or immunostained membranes was done using GelAnalyzer 23.1 (http://www.gelanalyzer.com).

### Protection of susceptible bacteria against IPM action by *A. baumannii* OMVs

Previously described microbiological procedures were employed for this purpose (6, 86). Briefly, Mueller-Hinton (MH) agar plates (Difco) were inoculated with liquid cultures of susceptible indicator bacterial strains, including *A. baumannii* ATCC 17978 and *Escherichia coli* ATCC 25922, previously adjusted to a McFarland standard turbidity of 0.5, by spreading the indicator bacteria on the agar surface. Then, 10 µg IPM-containing disks (BD BBL Sensi-Disc) were placed in the center of the agar surface, followed by four filter disks equidistantly placed from the antibiotic disk within the expected IPM growth inhibition zone. The peripheral disks were loaded, unless indicated in the legend to figures, with 20 µL each of Disk 1, PBS; disk 2, PBS + 0.1% vol/vol Triton X-100, disk 3, OMV suspension; disk 4: OMV suspension + 0.1% vol/vol Triton X-100, respectively. The non-ionic detergent Triton X-100 was used to disrupt the OMV structure, exposing the internal lipo-OXA-58 CHDL to IPM. After overnight incubation of the agar plates at 37°C, IPM inactivation was evaluated by comparing the growth of susceptible indicator bacteria near the disks within the IPM inhibition halos.

### Accessibility of OMV-associated proteins to proteinase K

Purified OMVs from ATCC17978 cells indicated in the Legend to Figures were treated at 37°C with 0.05 U/mL *Tritirachium album* proteinase K immobilized in agarose beds (Proteinase K-Agarose, Merck). This procedure is designed to digest OMV proteins exposed to the external medium and any bound exogenous proteins product of cell lysis that adhered to the vesicles during preparation (85). Samples were collected at different time points, treated with 1 mM PMSF to inactivate proteinase K, and analyzed by SDS-PAGE, as described above. Parallel assays were also performed in the presence of 0.1% (wt/vol) SDS to expose both externally associated proteins and the internal OMV proteins to proteinase K activity.

### Determination of plasmid copy number (PCN) of *bla*_OXA-58_-carrying plasmids in ATCC17978 transformants

Real-time quantitative PCR (RT-qPCR) (87) was used to determine the difference in threshold cycle value (ΔC_T_) between the plasmid single-copy *bla*_OXA-58_ gene (target) and the *A. baumannii* single-copy chromosomal *recA* gene (GenBank accession NZ_CP043953.1). Oligonucleotide primers were designed using Primer3 (https://primer3.ut.ee). The primer pair used for amplification of a 155 bp *recA* fragment spanning was recAFw: 5´-TACAGAAAGCTGGTGCATGG-3´ and recARv: 5´-TGCACCATTTGTGCCTGTAG-3´, and that for amplification of a 150 bp *bla*_OXA-58_ fragment was OXA-58Fw: 5´-TAGAGCGCAGAGGGGAGAAT-3´ and OXA-58Rv: 5´-CATCACCAGCTTTCATTTGC-3´. Total DNA was extracted from the analyzed ATCC17978 transformants by resuspending cell colonies grown in LB agar in 50 µL of autoclaved distilled water, followed by a 10 min incubation at 95°C. After cooling in an ice-water bath, the suspension was cleared by centrifugation, and the DNA in the supernatants was quantified using a NanoDrop ND-2000 Microvolume Spectrophotometer (Nanodrop Technologies, Delaware). RT-qPCR assays were performed with at least three independent 10-fold DNA dilution series for each ATCC17978 transformant tested. RT-qPCRs were conducted using a StepOne Real-Time PCR System (Applied Biosystems, Foster City, CA) in separate assays for the amplification of chromosome- or plasmid-specific genes. The mixtures contained, per assay, 1 x HOT FIREPol EvaGreen qPCR Mix Plus (ROX, Solis BioDyne, Estonia), 2–20 ng total genomic DNA, 2.5 mM MgCl_2_, 0.15 µM of each oligonucleotide primer, and PCR-quality distilled H_2_O for a total volume of 20 µL. Amplification conditions included an initial 10 min denaturation step at 95°C, followed by 40 cycles of 15 s at 95°C, 30 s at 62°C, and 20 s at 72°C. This was followed by melting curve analysis with a temperature gradient of 0.1°C/s from 70 to 95°C to analyze amplification specificity. Data analysis and C_T_ calculations were performed using the StepOne Software v2.1 (Applied Biosystems), and the PCN values are expressed as (1 + *E*)^ΔCT^ (87), where *E* is 0.95 for a determined PCR amplification factor of 95%, and ΔC_T_ the difference in mean threshold cycle values of the *bla*_OXA-58_ amplicon and the *recA* reference amplicon in the analyzed ATCC17978 transformant.

### Determination of growth parameters

Growth curves of *A. baumannii* ATCC17978 or *E. coli* DH5α cells transformed with the plasmids indicated in the corresponding Legends to Figures were determined using Microtiter Plate Readers (Synergy HTX, BioTek) following described protocols (88). For *A. baumannii*, growth rates are reported as the first-order growth rate constant, the slope of the least-squares fit of ln(DO600) versus time, in h^-1^ units. The values are indicated as the mean rates ± 95% confidence interval, based on a minimum of four independent growth curves. The lag time was estimated by extrapolating the linear portion of the curve back to the initial Abs, i.e., the time to enter the exponential phase (88). For *E. coli* DH5α, the cells were grown at 30°C in LB medium supplemented with 50 µg/mL Km until an Abs_600nm_ of 0.5. At this stage, arabinose (0.01%, 0.001%, or 0.0001% wt/vol) was added to the cultures, which were further incubated under similar conditions while Abs_600_ was recorded.

To evaluate OXA-58 production in *E. coli* DH5α, the cells were grown in LB liquid medium supplemented with 50 µg/mL Km and 0.0001% (wt/vol) arabinose, as described above. After an overnight incubation, 1 mL of each culture was subjected to centrifugation at 14,000 rpm for 10 min at 4°C, and the harvested cells were resuspended in SDS loading buffer and subjected to SDS-PAGE and immunoblot assays using α-OXA-58 antibodies as described above.

### Bioinformatic analyses

SignalP-6.0 (89) (https://services.healthtech.dtu.dk/services/SignalP-6.0/) was used for predictions of the nature of the N-terminal signal peptides and the location of signal peptidases I or II (SPaseI and SPaseII, respectively) cleavage sites on the OXA-58 and OXA-58C19A precursors. Amino acid sequence alignments were performed using the function “build” function of ETE3 3.1.2 implemented on GenomeNet (https://www.genome.jp/tools/ete/).

## SUPPLEMENTAL MATERIAL

Supplemental material aac.01099-25-s0001.pdf Fig. S1 to S6.

ASM does not own the copyrights to Supplemental Material that may be linked to, or accessed through, an article. The authors have granted ASM a non-exclusive, world-wide license to publish the Supplemental Material files. Please contact the corresponding author directly for reuse.
