# Supplementary material for: The lipoprotein biosynthesis pathway: key to OXA-mediated carbapenem resistance in Acinetobacter baumannii
Source: Antimicrob Agents Chemother. 2025 Nov 4;69(12):e01099-25. doi: 10.1128/aac.01099-25 (PMC12691667; doi:10.1128/aac.01099-25)
Supplement: Supplemental material — Fig. S1 to S6. [file aac.01099-25-s0001.pdf]

SUPPLEMENTAL MATERIAL

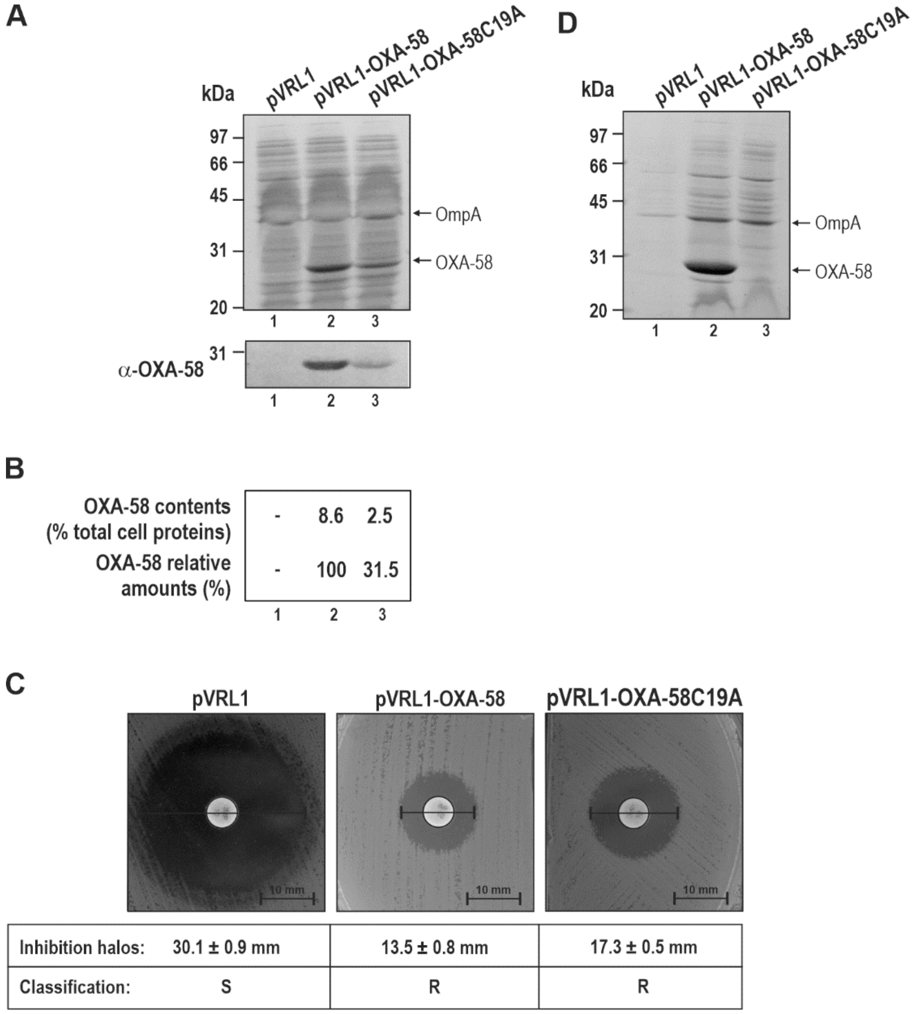

Figure S1. Expression of OXA-58 and OXA-58C19A in ATCC17978 cells directed by pVRL1-based plasmids. A) SDS-PAGE and immunoblot analysis using α-OXA-58 of whole cell extracts of ATCC17978 cells carrying: lanes 1, pVRL1 (empty vector); lanes 2, pVRL1-OXA-58; lanes 3, pVRL1-OXA-58-C19A. The final positions of the OXA-58 (WT) and OXA-58v (mutant) in the gels are indicated at the right margin. **B)** OXA-58 quantification as determined from densitometric analysis of the Coomassie-Blue stained SDS-gels in combination with the immunoblot analysis. See legend to Fig. 2 for details. **C)** IPM inhibition halos determined on Mueller-Hinton agar obtained for ATCC17978 cells transformed with the indicated plasmids. Classification in susceptible (S) or IPM-resistant (R) is based on CLSI standards for *Acinetobacter* clinical isolates. **D)** SDS-PAGE analysis of purified OMV from ATCC17978 cells carrying the indicated plasmids. OXA-58 and OmpA positions in the gels (determined by immunoblot analyses, not shown) are indicated.

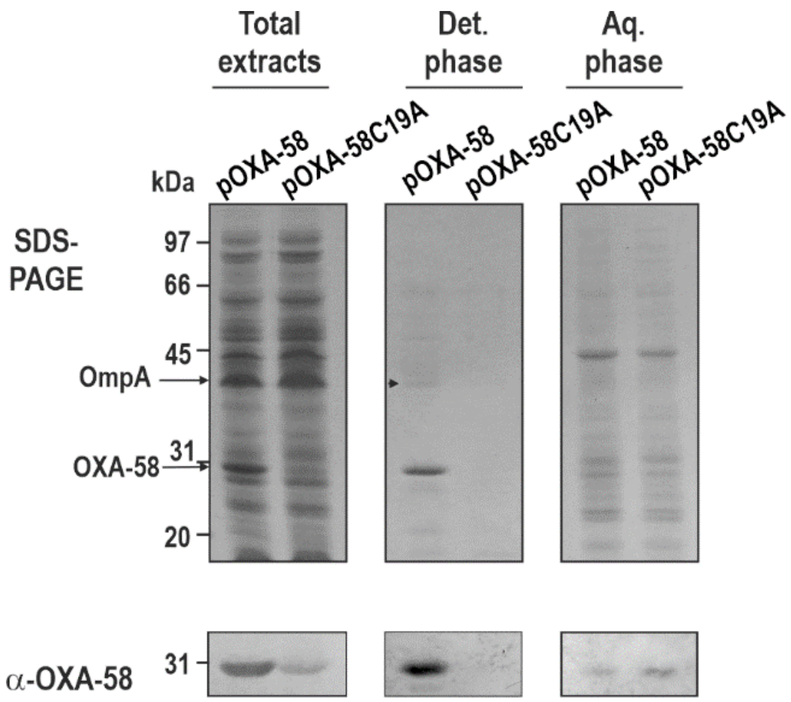

Figure S2. Triton X-114 fractionation evidences the hydrophobic nature conferred by lipidation to *A. baumannii* OXA-58. SDS-PAGE analyses of total cell extracts (Total extracts), detergent (Det. phase) and aqueous (Aq. phase) phases obtained after Triton X-114 fractionation of total extracts of ATCC17978 cells expressing OXA-58 (pOXA-58) or OXA-58v (pOXA-58C19A). The immunoblot analysis using α-OXA-58 antibodies is shown at the bottom. Fractionation was performed as described in Materials and Methods. OXA-58 and OmpA final positions in the gels are indicated by arrows or an arrowhead at the left margins.

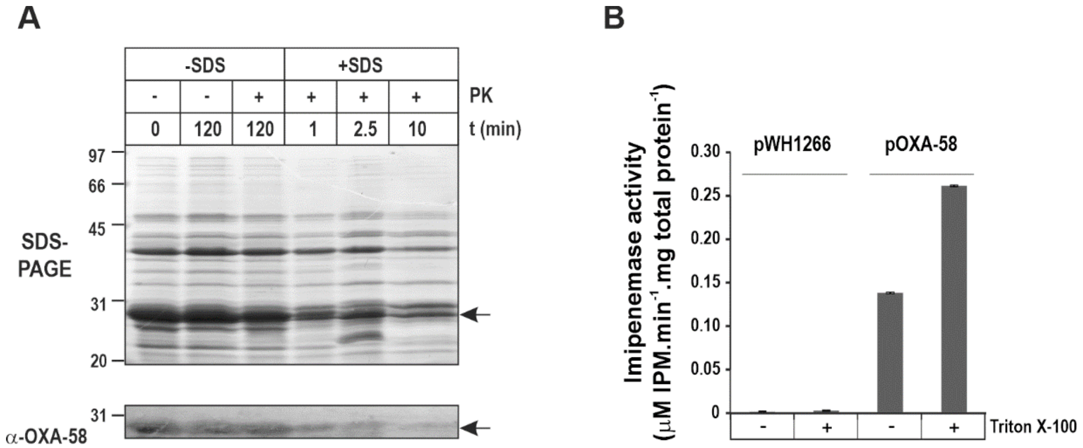

Figure S3. Lipidated OXA-58 is secreted in *A. baumannii* inside OMV. A) Proteinase K protection assay of OMV from ATCC17978/pOXA-58 cells. OMV were treated with proteinase K-agarose. Samples were collected at indicated times, treated with 1 mM phenylmethylsulfonyl fluoride (PMSF), and analyzed by SDS-PAGE (top) and immunoblotting (α-OXA-58, bottom). Parallel assays were performed without (-SDS) or with (+0.1% w/v) SDS to expose internal OMV proteins to proteinase K. OXA-58 position in the gel is indicated with an arrow at the right margin. **B)** Imipenemase activity of OMV obtained from ATCC17978/pWH1266 or ATCC17978/pOXA-58 cells before (-) and after (+) 0.1% v/v Triton X-100 treatment.

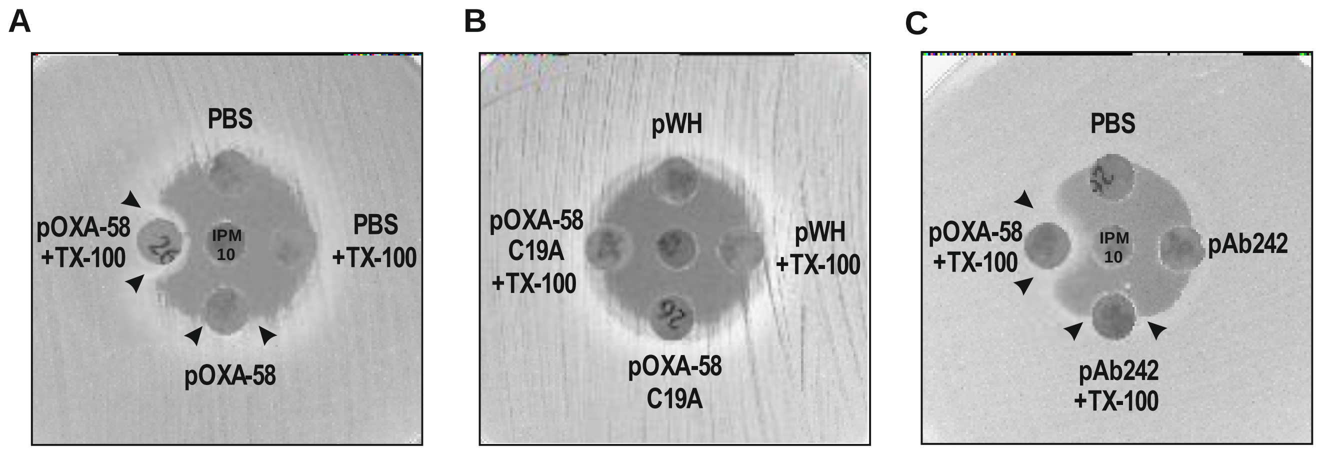

Figure S4. Protection of susceptible *A. baumannii* ATCC17978 cells from IPM action by OMV. The effects of OMV from ATCC17978 cells carrying pOXA-58, pOXA-58-C19A, pAb242_25, or the pWH1266 plasmid vector (pWH) on an underneath layer of growing susceptible bacteria were tested using a previously described microbiological procedure (1). Briefly, Mueller-Hinton (MH) agar plates were first inoculated with a standardized inoculum (0.5 McFarland turbidity) of susceptible ATCC17978 cells. A 6 mm disc containing 10 μg IPM was placed at the center, and 4 peripheral discs at the periphery within the expected inhibition zone. The latter were loaded with 10 μl of 5 μg total OMV protein from the indicated samples. **A)** bottom disc, OMV isolated from ATCC17978/pOXA-58 cells (pOXA-58). Left disc, same, supplemented with 0.1 % (v/v) Triton X-100 (pOXA-58+TX-100). Upper disc, 10 μl phosphate-buffer saline (PBS). Right disc, 10 μl PBS plus 0.1 % (v/v) Triton X-100 (PBS+TX-100). **B)** bottom disc, OMV from ATCC17978/pOXA-58C19A cells (pOXA-58C19A). Left disk, same, supplemented with 0.1 % (v/v) Triton X-100 (pOXA-58+TX-100). Upper disk, OMV from ATCC17978/pWH1266 cells (pWH). Right disk, same, supplemented with 0.1 % (v/v) Triton X-100 (pWH+TX-100). **C)** right disc, OMV isolated from ATCC17978/pAb242 cells (pAb242). Bottom disk, same, supplemented with 0.1 % (v/v) Triton X-100 (pAb242 +TX-100). Left disc, OMV from ATCC17978/pOXA-58 cells supplemented with 0.1 % (v/v) Triton X-100 as in **(A)**. Upper disk, 10 μl PBS. After an overnight incubation at 37°C, OMV protection against IPM inhibition of growth of the susceptible indicator bacteria was observed near the discs within the inhibition halos. Closed arrowheads highlight different levels of protection.

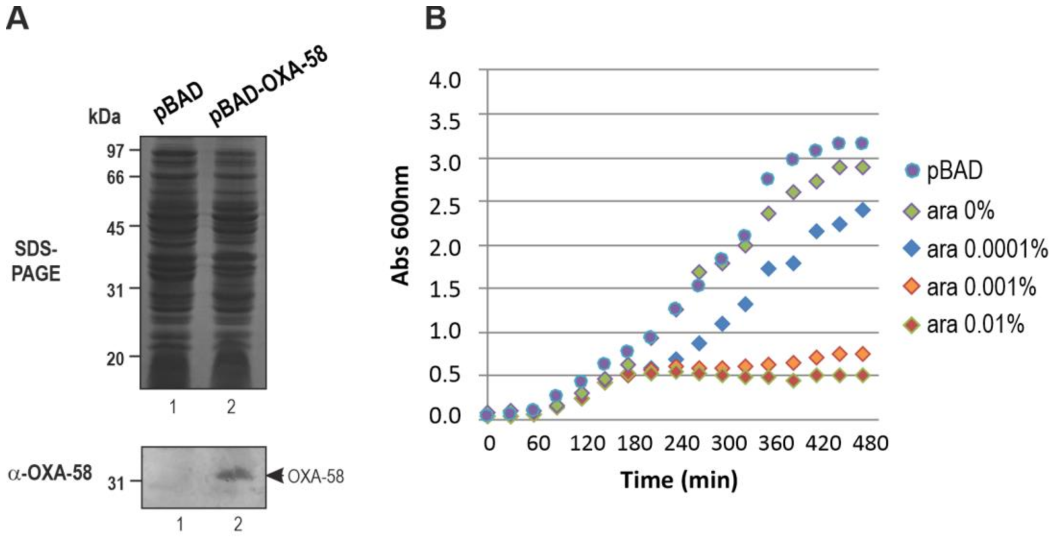

C

DH5α cells carrying	MIC IPM (μg/ml)	MIC Amp (μg/ml)
pBAD (empty vector)	0.125	4
pBAD-OXA-58	0.125	16

Figure S5. pBAD-directed production of pre-proOXA-58 in *E. coli* DH5α cells. A) SDS-PAGE (upper) and immunoblot (lower) analyses of total cell extracts of *E. coli* DH5α cells transformed with: pBAD empty vector (lane 1); pBAD-OXA-58 (lane 2). The cells were grown in LB medium supplemented with 0.0001 % w/v arabinose. **B)** Growth curves of DH5α cells transformed with pBAD-OXA-58, in the presence of the indicated arabinose (ara) concentrations in w/v**. C)** MIC values for imipenem (IPM) and ampicillin (Amp) of the cells described in (A). For details of pBAD-OXA-58 construction see Materials and Methods.

```
                                                           α1             β1
                                                    aaaaaaaaaaaaaa   bbbbbbb
                                                                          60
ABOXA-58        MKLLKILSLVCLSISIGACAEHSMSRAKTSTIPQVNNSIIDQNVQALFNEISADAVFVTY
ABOXA-23        ----MNKYFTCYVVASLFLSGCTVQHNLINETPSQIVQGHNQVIHQYFDEKNTSGVLVIQ
ABOXA-24        --MKKFILPIFSISILVSLSACSSIKTKSEDNFHISSQQHEKAIKSYFDEAQTQGVIIIK
ABOXA-51        ---MNIKTLLLITSAIFISACSPYIVTANPNHSASKSDEKAEKIKNLFNEVHTTGVLVIQ
                                                         : ::  *:*  : .*::

                       β2     α2   β3         α3                β′          3_10 β″
                b   bbbbbb aaaaa bbb aaaaaaaaaaaaaaa       bbbb      aaaaabb
                                                                         120
ABOXA-58        DGQNIKKYGTHLDRAKTAYIPASTFKIANALIGLENHKATSTEIFKWDGKPRFFKAWDKD
ABOXA-23        TDKKINLYGNALSRANTEYVPASTFKMLNALIGLENQKTDINEIFKWKGEKRSFTPWEKD
ABOXA-24        EGKNLSTYGNALARANKEYVPASTFKMLNALIGLENHKATTNEIFKWDGKKRTYPMWEKD
ABOXA-51        QGQTQQSYGNDLARASTEYVPASTFKMLNALIGLEHHKATTTEVFKWDGQKRLFPEWEKD
                 .:. . **. * ** . *:******: *******::*:  .*:***.*: * :  *:**

                β″    α4         α5          α6                   α7    β4   α8
                Bbaaaaaaa  aaaaaaaaaaa aaaaaaaaaaaa           aaaaaa bbb aaa
                                                                         180
ABOXA-58        FTLGEAMQASTVPVYQELARRIGPSLMQSELQRIGYGNMQIGTEVDQFWLKGPLTITPIQ
ABOXA-23        MTLGEAMKLSAVPVYQELARRIGLDLMPKEVKRIGFRNAEIGPPVENFWFGRPLKVTPIQ
ABOXA-24        MTLGEAMALSAVPVYQELARRTGLELMQKEVKRVNFGNTNIGTQVDNFWLVGPLKITPVQ
ABOXA-51        MTLGDAMKASAIPVYQDLARRIGLELMSKEVKRVGYGNADIGTQVDNFWLVGPLKITPQQ
                :***:**  *::****:**** * .** .*::*:.: * :**  *::**:  **.:** *

                  α8                  α9      β5          β6            β7
                aaaaaaaaaa      aaaaaaaaaa bbbbb  bbbbbbbbbb    bbbbbbbbbb
                                                                         240
ABOXA-58        EVKFVYDLAQGQLPFKPEVQQQVKEMLYVERRGENRLYAKSGWGMAVDPQVGWYVGFVEK
ABOXA-23        EVEFVSQLAHTQLPFREKVQANVKNMLLLEESNGYKIFGKTGWARDIKPQVGWLTGWVEQ
ABOXA-24        EVNFADDLAHNRLPFKLETQEEVKKMLLIKEVNGSKIYAKSGWGMGVTPQVGWLTGWVEQ
ABOXA-51        EAQFAYKLANKTLPFSPKVQDEVQSMLFIEEKNGNKIYAKSGWGWDVDPQVGWLTGWVVQ
                *.:*. .:*:  ***  :.* :*:.** ::. .  :::.*:**.  : ***** .*:* :

                         β8                 α10
                    bbbbbbbbbb      aaaaaaaaaaaaaa
                                                     280
ABOXA-58        ADGQVVAFALNMQMKAGDDIALRKQLSLDVLDKLGVFHYL
ABOXA-23        PDGKIVAFALNMEMRSEMPASIRNELLMKSLKQLNII---
ABOXA-24        ANGKKIPFSLNLEMKEGMSGSIRNEITYKSLENLGII---
ABOXA-51        PQGNIVAFSLNLEMKKGIPSSVRKEITYKSLEQLGIL---
                .:*: :.*:**::*:     ::*:::  . *.:*.::
```

Figure S6. Sequence alignments of *A. baumannii* OXA-58, OXA-23, OXA-24 and OXA-51 precursor proteins. The typical motifs of class D β-lactamases: STFK, (F/Y)GN, and K(S/T)G, are underlined below the alignments. Identical amino acids at a given position are also indicated below the alignments with asterisks (*), conservative substitutions with dots (:), and indels (insertions and deletions) with hyphens. The variable N-terminal region encompassing the lipoprotein transit peptide and connector sequences are indicated in bold. The lipoboxes are underlined in each sequence. The numbers above the rows refer to the last amino acid residue

in OXA-58. The alignments were done using CLUSTAL 2.1 (https://www.genome.jp/tools/ete/). The elements of secondary structure, as determined by crystallographic analysis (2), are also indicated above the sequences. GeneBank accession numbers: OXA-58: AAW57529.1, *A. baumannii* strain MAD; OXA-23: CAB69042.1, *A. baumannii* strain 6B92; OXA-24/40: CAB92323.2, *A. baumannii* strain RYC 52763/97; OXA-51 (OXA-259): QKY23678.1, *A. baumannii* strain ATCC 17978.

References

1. Marchiaro P, Ballerini V, Spalding T, Cera G, Mussi MA, Morán-Barrio J, Vila AJ, Viale AM, Limansky AS. 2008. A convenient microbiological assay employing cell-free extracts for the rapid characterization of Gram-negative carbapenemase producers. J Antimicrob Chemother 62:336–344.
2. Smith CA, Antunes NT, Toth M, Vakulenko SB. 2014. Crystal structure of carbapenemase OXA-58 from Acinetobacter baumannii. Antimicrob Agents Chemother 58:2135–2143.
